# Wire Ropes and CFRP Strips to Provide Masonry Walls with Out-Of-Plane Strengthening

**DOI:** 10.3390/ma12172712

**Published:** 2019-08-24

**Authors:** Elena Ferretti

**Affiliations:** Department of Civil, Environmental and Materials Engineering—DICAM, Alma Mater Studiorum Università di Bologna, 40136 Bologna, Italy; elena.ferretti2@unibo.it; Tel.: +39-051-209-35-15

**Keywords:** masonry buildings, hammering actions, out-of-plane strengthening, three-dimensional strengthening, carbon fiber reinforced polymer (CFRP) strips, textile reinforced mortar (TRM)

## Abstract

The present paper deals with an improvement of the strengthening technique consisting in the combined use of straps—made of stainless steel ribbons—and CFRP (Carbon Fiber Reinforced Polymer) strips, to increase the out-of-plane ultimate load of masonry walls. The straps of both the previous and the new combined technique pass from one face to the opposite face of the masonry wall through some holes made along the thickness, giving rise to a three-dimensional net of loop-shaped straps, closed on themselves. The new technique replaces the stainless steel ribbons with steel wire ropes, which form closed loops around the masonry units and the CFRP strips as in the previous technique. A turnbuckle for each steel wire rope allows the closure of the loops and provides the desired pre-tension to the straps. The mechanical coupling—given by the frictional forces—between the straps and the CFRP strips on the two faces of the masonry wall gives rise to an I-beam behavior that forces the CFRP strips to resist the load as if they were the two flanges of the same I-beam. Even the previous combined technique exploits the ideal I-beam mechanism, but the greater stiffness of the steel wire ropes compared to the stiffness of the steel ribbons makes the constraint between the facing CFRP strips stiffer. This gives the reinforced structural element a greater stiffness and delamination load. In particular, the experimental results show that the maximum load achievable with the second combined technique is much greater than the maximum load provided by the CFRP strips. Even the ultimate displacement turns out to be increased, allowing us to state that the second combined technique improves both strength and ductility. Since the CFRP strips of the combined technique run along the vertical direction of the wall, the ideal I-beam mechanism is particularly useful to counteract the hammering action provided by the floors on the perimeter walls, during an earthquake. Lastly, when the building suffers heavy structural damage due to a strong earthquake, the box-type behavior offered by the three-dimensional net of straps prevents the building from collapsing, acting as a device for safeguarding life.

## 1. Introduction

This paper is part of a research project on improving the out-of-plane behavior of masonry walls by combining different strengthening techniques [1,2,3,4]. The key idea of the combined technique is to exploit friction in order to achieve a mechanical coupling between different strengthening devices. Due to the mechanical coupling, the strengthening devices work together giving rise to a new resistant mechanism, with strengthening characteristics not owned by any of the constituent strengthening devices. Consequently, the resistant mechanism of the combined technique is not a combination of the main features of the constituent techniques.

**Figure 1:**
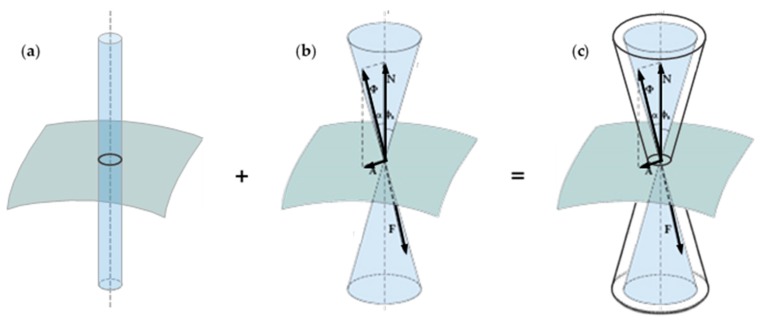
How the addition of a physical bond–represented by (**b**) the conical limit surface of static friction–changes the limit surface in static conditions from (**a**) the cylindrical surface of the chemical bond to (**c**) the double truncated cone of the cohesive static friction [1].

Specifically speaking, the combined technique proposed in [1,4] makes use of FRP (Fiber Reinforced Polymer) strips tied by stainless steel straps. The friction at the interface between FRP strips and masonry walls provides a physical bond that overlaps with the chemical bond given by the resin alone. As shown in Figure 1, this modifies the limit surface of the interface bond—which becomes a cohesive physical bond—allowing the FRP strips to withstand higher shear forces before delaminating from the masonry wall under bending loads. This increases the delamination load of the FRP strip on the tensioned side of the bent wall.

**Figure 2:**
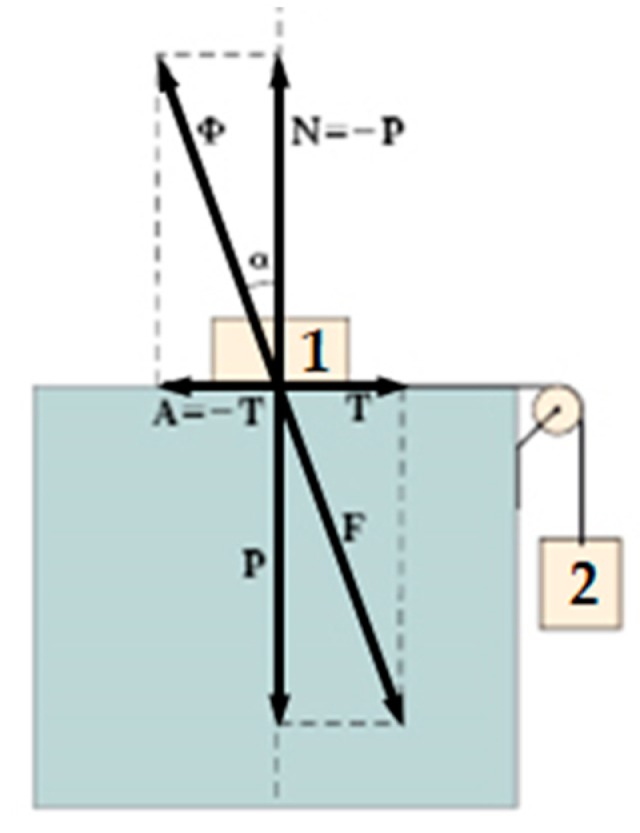
Body 1 at rest under the combined action of its weight force and the shear force provided by body 2: Active and reactive forces developed on the support plane.

As far as the active and reactive forces in Figure 1 are concerned:

- **N** is the normal force that develops as a reaction to the weight force **P**, exerted by the body at rest in Figure 2 (body 1): **N** is equal and opposite to **P**;
- **A** is the frictional force that develops as a reaction to the shear force **T**, exerted by the hanging body in Figure 2 (body 2): **A** is equal and opposite to **T** as long as body 1 is at rest;
- **Φ** is the resultant of the active forces, **N** and **A**, and is applied to body 1;
- **F** is the resultant of the reactive forces, **P** and **T**, and is applied to the support plane.

The angle between **Φ** and its component vector **N** is $\alpha =\tan^{-1}A/N$. Furthermore, the aperture of the cone of static friction is twice the angle of static friction, $\phi_{s}$, which, assuming to eliminate body 2 and progressively incline the support plane of Figure 2, measures the maximum inclination angle that allows body 1 not to slide on the inclined plane.

The frictional force **A** develops as a reaction force to counteract the relative sliding at the interface between two bodies even when the normal force **P** is not the weight force. Therefore, to take advantage of the beneficial effect of friction, it is necessary to press the FRP strips and the masonry wall together. The device used at an early stage of the research program to press the FRP strips and the masonry wall together is the CAM (Active Confinement of Masonry) system [5,6,7,8,9,10,11,12], a continuous three-dimensional net of straps that post-compress the wrapped masonry by means of a pre-tension of the straps. The pre-tension makes the CAM system an active strengthening system. Specifically, the CAM system belongs to the category of strengthening of “horizontal and vertical ties” [13,14]. This category is particularly useful in cases of the lack of transversal links and ineffective connections between walls or between walls and floors, as is usual in historical buildings [6,7,15,16,17,18,19,20,21,22,23,24,25,26,27,28,29].

**Figure 3:**
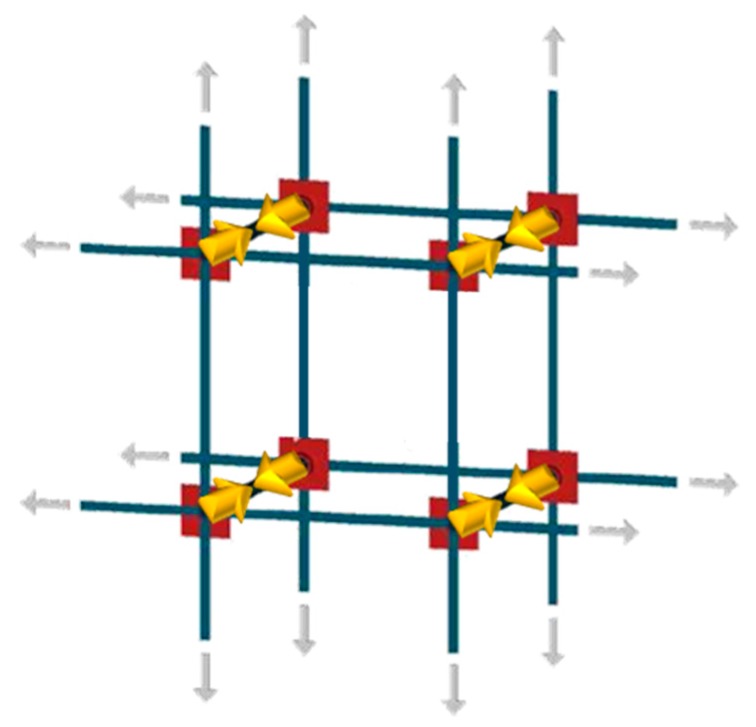
Nodal forces transferred by the CAM straps: Balanced forces in gray and not balanced forces in yellow [1].

The main target of the CAM system, patented in 1999 by Dolce and Marnetto, is to provide the wrapped masonry with an additional state of hydrostatic stress, which increases the safety factor of the masonry building. Nevertheless, a more accurate analysis of the actual mechanism for transferring stresses from the straps to the nodes of the masonry units [2,3] showed that the CAM system does not actually provide a hydrostatic state of stress. In the specific case of a rectangular CAM net, for instance, the straps act in the plane of the wall with pairs of equal and opposite nodal forces, while the nodal forces in the wall thickness are not counteracted (Figure 3). Consequently, the rectangular retrofitting system does not transfer any in-plane force, while it provides an out-of-plane compression to the nodes.

By providing compression forces only along the thickness of the wall, the straps of the CAM system, deprived of their original use, find a new employment in the combined technique as devices to push the FRP strips against the masonry wall. In fact, if applied over the FRP strips, the pre-tensioned straps act on the FRP strips in the same way that **P** compresses body 1 against its support plane in Figure 2. On the stretched side of a bent wall, this modifies the interface bond of the FRP strips from a chemical to a cohesive physical bond (Figure 1), increasing the delamination load by means of the frictional effect.

**Figure 4:**
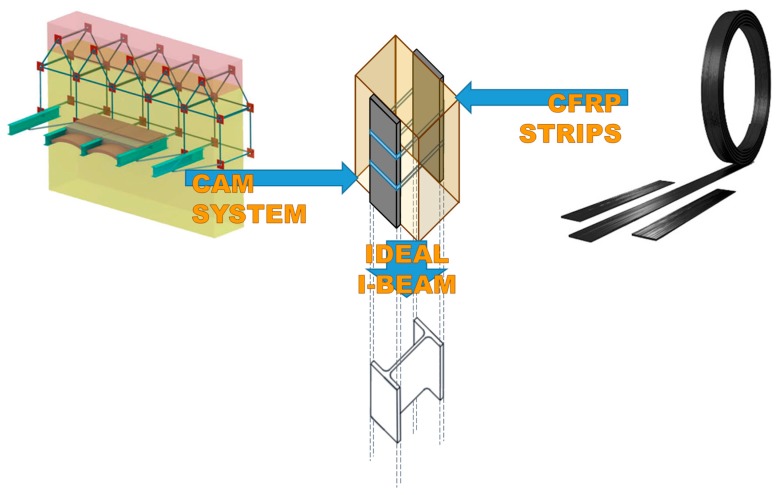
The transverse connections between the FRP strips give rise to an I-beam behavior [1].

**Figure 5:**
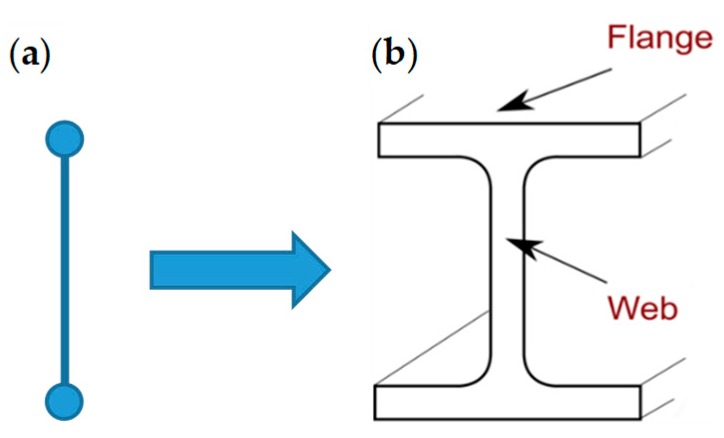
Concept of the straps/strips combined technique: Two FRP strips tied by CAM ribbons behave as (**a**) two mass points connected by a stiffness constraint, which is the ideal scheme of behavior of (**b**) a bent I-beam in its cross-section.

A useful application of the straps/strips combined technique consists in placing the FRP strips vertically, one in front of the other on the two faces of a masonry wall (Figure 4). In this case, an additional advantage in terms of reinforcement overlaps with the benefits of the cohesive physical bond on the stretched side: When tied by the CAM straps as in Figure 4, the two FRP strips work together due to the transverse connection established by the CAM system (Figure 5). The resulting strengthening effect under bending loads is the same as that provided by a bracing FRP I-beam, which acts as an embedded buttress without having to cut the masonry wall to insert it.

The strengthening effect of the embedded buttress is the more remarkable the thicker the masonry wall, because a greater wall thickness increases the web length (Figure 5) and, consequently, the moment of inertia of the ideal I-beam [4]. Therefore, the ideal I-beam increases the out-of-plane strength much more than the individually taken FRP strips.

Furthermore, the floors of a multi-story building do not interrupt the continuity of the ideal I-beam thanks to the ability of the CAM net to cross the floors easily [1]. Consequently, during a seismic event orthogonal to a series of walls in the multi-story building, the ideal I-beam is able to counteract the hammering action of the floors on the perimeter walls. Since the out-of-plane failure of URM (Unreinforced Masonry) walls is the main cause of the catastrophic collapses of URM buildings, this latter feature is of paramount importance to avoid serious loss of human lives during an earthquake.

The use of buttresses is one of the most ancient techniques developed over the centuries for the retrofit/strengthening of URM structures. In its original use, where buttresses were structures built against a wall, this ancient technique is effective but highly invasive and causes great increases in mass. As a result, with the advent of new technologies, buttresses were gradually abandoned in favor of more recent strengthening techniques, some of which are base isolation, seismic dampers, surface treatments, mortar joint treatments, external steel reinforcement, post-tensioning, mesh reinforcement, reticulatus system, confinement with ring beams, tie bars, and fiber/textile-reinforced mortar [2,30,31,32]. The straps/strips combined technique recuperates the simple strengthening scheme of the buttress, but minimizing invasiveness and mass increases.

**Figure 6:**
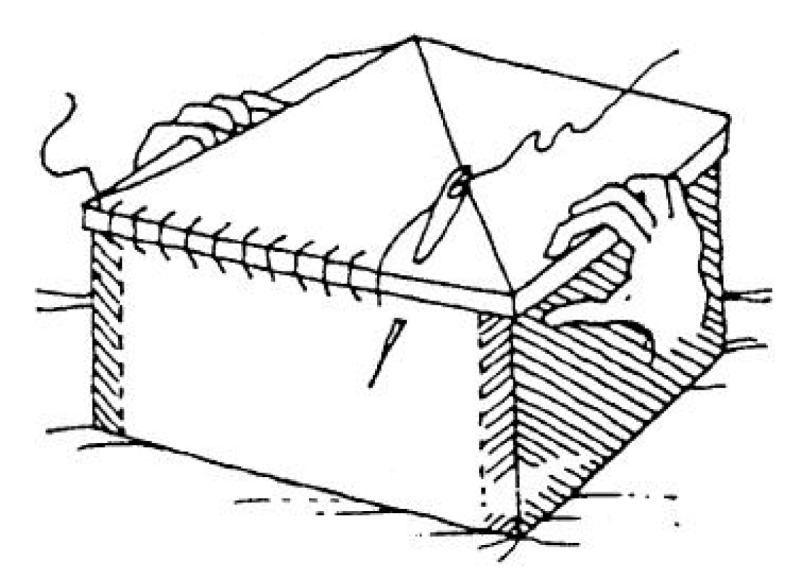
The effectiveness of structural connections provides box-type behavior to the building.

The ability of the combined technique to provide an out-of-plane cross-bracing of walls in masonry buildings is all the more remarkable precisely because it allows us to obtain the same strengthening mechanism as an embedded buttress without a significant increase in the mass of the building. In fact, any increase in mass is particularly harmful for the building, because it increases the attraction of seismic forces. Lastly, by obtaining the same effect as a buttress with the use of the CAM system, it is possible to enrich the out-of-plane cross-bracing device with characteristics not owned by either traditional buttresses or the most recent strengthening techniques. In fact, the structural connections provided by the straps of the CAM system allows the combined technique to guarantee the so-called box-type behavior [33,34,35], which consists in tying the building elements to each other, starting from the foundation (Figure 6). This leads to a series of structural improvements, one of the most significant of which is the reduction of out-of-plane wall failures.

## 2. The Straps/Strips Combined Technique

**Figure 7:**
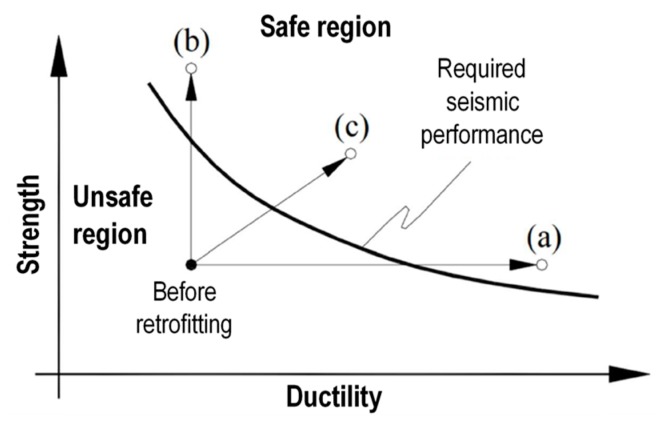
Comparison between retrofitting systems: An FRP reinforcement leads the structure to point (**b**), increasing strength but not ductility; a three dimensional reinforcement with steel ribbons leads the structure to point (**a**), increasing ductility but not strength; the combined technique leads the structure to point (**c**), increasing both strength and ductility.

As detailed in Section 1, the straps/strips combined technique is an out-of-plane strengthening technique that developed as an improvement of the CAM system. Similar to the CAM system:

- It consists of a three-dimensional continuous strengthening system that leads to a box-type behavior of the retrofitted building (Figure 6).
- It establishes good transversal connections, which are particularly useful in cases of multi-layer masonry walls with weak connections between the vertical layers.
- It makes use of stainless steel straps that avoid the occurrence of corrosion [36] and compatibility [37] problems.
- It allows the straps to form closed loops that cross the thickness of the masonry wall.
- It is an active reinforcement technique, since the fastening system provides a pre-tension to the straps. Thanks to the pre-tension, the straps do not require any damage to begin to post-compress the masonry enclosed within them.
- It makes use of special protective elements at the loop corners, to avoid damage due to concentration of stresses at the corners.
- It is easily concealable under a plaster layer because the thicknesses of the straps and the protective elements are of the same order of magnitude as the thickness of the plaster. Therefore, from an aesthetic point of view it is minimally invasive.
- It overcomes the irregularities of the walls easily, making it possible to strengthen even ornamented or complex-shaped walls.
- It minimizes the increase in the total weight of the structure, making it possible to avoid further attraction of seismic forces.
- It continues to wrap the wall even after masonry crushing, allowing the damaged building not to collapse. This high degree of ductility (Figure 7) allows the combined technique to survive structural damage, acting as both a reinforcement system and a protection device.

The combined technique also inherits the ability to increase the out-of-plane maximum load of the masonry wall from the technology of composite materials (Figure 7). This is essential to improve the strength to the hammering actions of the masonry wall.

It is worth noting that the mechanical coupling gives rise to a new strengthening mechanism, which is not simply the result of the joint use of the two constituent strengthening techniques. In particular, the beam-like mechanism of the combined technique guarantees a residual load-bearing capacity higher than that provided by both the FRP strips and the straps.

To date, the experimental investigations on the combined technique made use of straps obtained from both steel ribbons—as for the CAM system—and steel wire ropes. Section 2.1 and Section 2.2 will deal with both possible techniques, namely, the first combined technique and the second combined technique.

### 2.1. First Combined Technique: Straps Made of Steel Ribbons

This combined technique is the first attempt to achieve a cross-bracing effect using steel straps and FRP strips. The technique inherits from the CAM system the use of stainless steel ribbons to make the straps, but the type of ribbons and the clamping system are not the same as those of the patented CAM system [1]. In particular, as far as the steel ribbons of the first combined technique are concerned, the strength was much lower and the ductility much greater, respectively, than the strength and ductility of the CAM straps [1].

**Figure 8:**
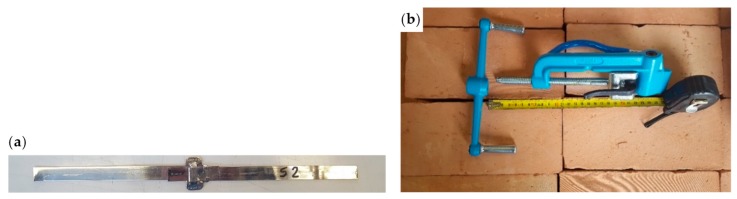
The fastening system of Reference [1]: (**a**) one fastened specimen for the characterization of the junctions and (**b**) the manual strapping tool used to fasten the steel ribbons.

Figure 8 shows a sealed ribbon and the manual device used to pre-tension the ribbons during strapping.

**Figure 9:**
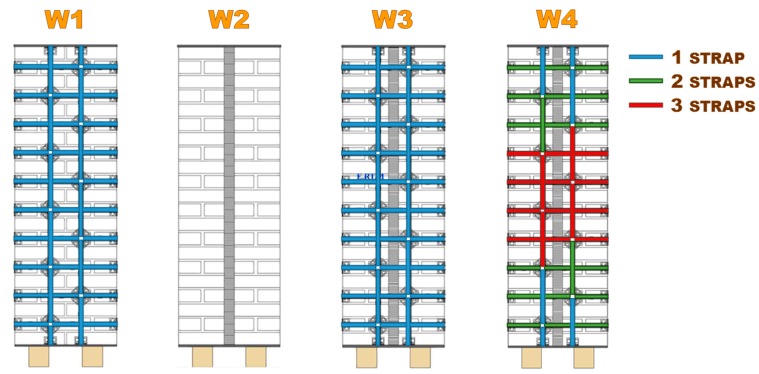
Arrangement of the straps for Specimens W1, W2, W3, and W4.

The authors of [1,4] showed the load/deflection diagrams in three-point bending for three wall specimens (Figure 9), strengthened by a net of steel ribbons (Specimen W1), two CFRP (Carbon Fiber Reinforced Polymer) strips (Specimen W2), and a net of steel ribbons and two CFRP strips (Specimen W3). Although the CAM system allows the use of up to four straps per loop, in specimens W1 and W3 there was only one strap per loop (Figure 9). After testing, the experimenters restored Specimen W3, modified the number of straps per loop as shown in Figure 9, and performed a further three-point bending flexural test on the restored specimen (Specimen W4). For details on the reason for the diversified number of straps per loop of Specimen W4, see [1].

All the bending tests took place under displacement control, after having overturned the specimens in horizontal configuration.

The purpose of these tests was to verify whether it is actually possible to obtain an I-beam behavior through the mechanical coupling of steel ribbons and CFRP strips. In this spirit, the tests involved only static actions, making it impossible to draw definitive conclusions for seismic actions.

**Figure 10:**
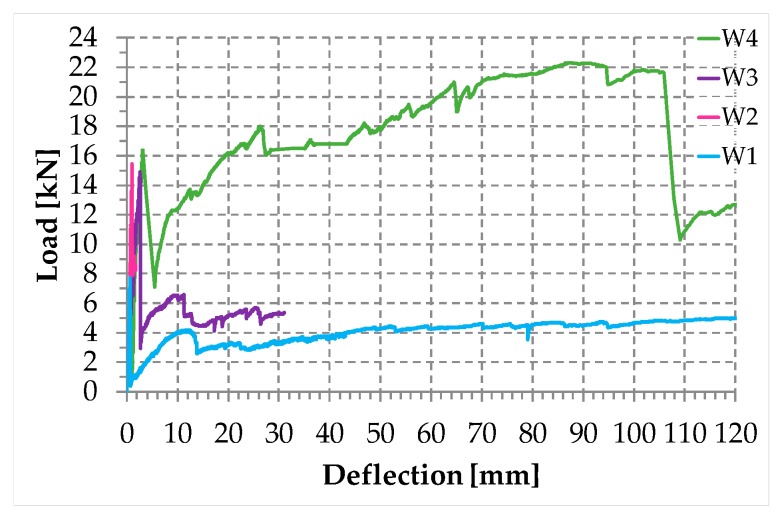
The load/deflection diagrams for the four specimens of the experimentation on the first combined technique.

During the flexural tests, some LVDTs (Linear Variable Differential Transformers) acquired the absolute displacements on the lower faces. The deflections in Figure 10 are the relative displacements in the central points of the lower faces, calculated as differences between the absolute displacements in the central points and the ends. The position of the instruments prompted the operator to interrupt the flexural test of Specimen W3 well in advance of Specimen W1 and Specimen W4. This is the reason why the load/displacement diagram for Specimen W3 is shorter than for Specimens W1 and W4.

The load/deflection diagrams in Figure 10 clearly show that both strength and ductility increase with the combined technique (Figure 7). In particular, for Specimens W3 and W4, the delamination loads are comparable to that of Specimen W2 (given only by the CFRP strips) and the post-peak ductility is comparable to that of Specimen W1 (given only by the steel straps). Actually, due to the high ductility, Specimens W1, W3, and W4 did not undergo a real collapse until the end of the test, which was necessary to avoid damage to the instrumentation. This means that the resilience of these specimens is extremely high: It recalls the resilience of FRP wrapped columns [38,39,40,41].

As already pointed out, the high ductility is associated with the ability of the CAM net to retain damaged material, protecting people from possible impact injuries. This is much more important than the ductility itself and is a value added of the combined technique.

The four peaks in Figure 10 return the load values for which the inner hinges open, with disconnection along a mortar bed joint. For Specimens W2, W3, and W4, they also return the loads of delamination. As far as Specimens W3 and W4 are concerned, the existence of post-delamination loads with values higher than those of Specimen W1 indicates that the steel straps retain the delaminated strips, allowing the I-beam mechanism to survive delamination, albeit with a deformable web (Figure 5). Actually, the stiffness of the transverse connection after delamination depends on the number of straps per loop. In fact, from the comparison between the post-delamination paths of Specimens W3 and W4, it follows that a greater number of straps per loop increases the post-delamination load-bearing capacity, which means that the web of the ideal I-beam (Figure 5) of Specimen W4 is stiffer than the web of Specimen W3.

It is precisely the post-peak behavior described above that allows us to affirm that the combined technique is something different from the joint use of the two constituent strengthening techniques. In fact, if the combined technique were a simple sum of the two constituent strengthening techniques, the loads along the post-delamination paths of Specimens W3 and W4 would not be greater than the loads along the post-delamination path of Specimen W1. On the contrary, by coupling two techniques created to provide a strengthening effect in the wall plane, the frictional forces caused by the mechanical coupling modify the strengthening mechanism from an in-plane to an out-of-plane mechanism (the ideal I-beam). This allows the combined technique not only to benefit from the main advantages offered by both the straps and the CFRP strips, but also to give a strengthening mechanism that is impossible to achieve with both strengthening techniques taken individually.

The increased number of longitudinal and transverse straps near to the middle cross-section of Specimen W4 (Figure 9) also decreases the load drop at delamination (Figure 10). This improves the overall behavior in real applications, since excessive load drops in the strengthened structural element can cause serious overloads in adjacent structural elements, which can collapse.

Actually, to avoid overloading phenomena, the load drop must not exceed 15–20% of the delamination load. Therefore, the residual load after delamination in Specimen W4 is still insufficient to prevent overload problems in the adjacent structural elements. However, since the stiffness of the used ribbons in very low, a residual load increase from 20% (Specimen W3) to 43% (Specimen W4) of the peak load is a good result, which still has room for improvement.

Furthermore, the post-delamination load of Specimen W4 is much greater than the post-delamination load of Specimen W3. The reason for the greater post-delamination load also lies in the greater number of steel straps, which increases the web stiffness of the ideal I-beam.

**Figure 11:**
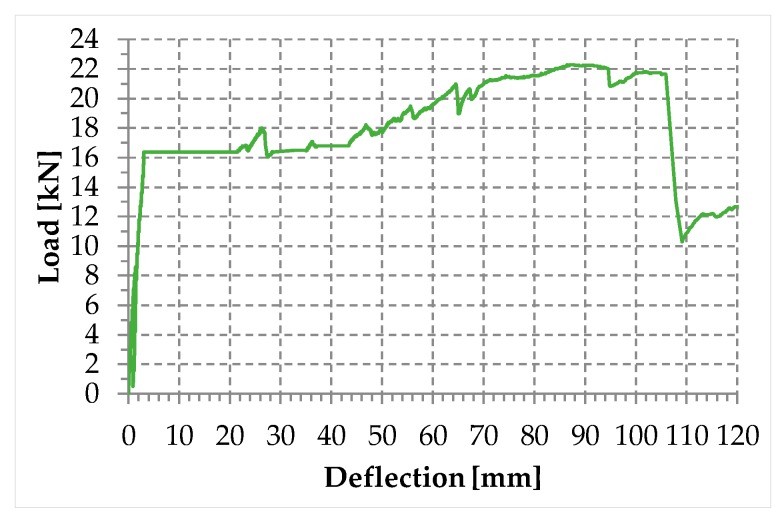
Load/deflection diagram for the first combined technique, in a hypothetical flexural test performed in the load control.

Since the post-delamination load of Specimen W4 increases up to recover and exceed the delamination load, the load/deflection diagram in the load control follows the horizontal path between the delamination load and the recovered load (Figure 11). The increase in load after the horizontal path of Figure 11 is of fundamental importance for the overall stability in real applications. In fact, as long as the increase in the displacement along the horizontal path is compatible with the resilience of the structure, the increase in the load beyond the recovered load avoids the redistribution of the load on the adjacent structural elements, which are no longer in danger of overloading.

In conclusion, the transverse straps have an effect on the delamination load and the load-bearing capacity after delamination, while the longitudinal straps have an effect on the load drop at the time of delamination. A greater number of ribbons also reduces the length of the horizontal path in Figure 11, so that the instantaneous deflection at the time of delamination can become compatible with the resilience of the structure in any case.

### 2.2. Second Combined Technique: Straps Made of Steel Wire Ropes

The second combined technique is an improvement of the first combined technique (Section 2.1). The improvement consists in using steel wire ropes for the straps, since the greater stiffness of the steel wire ropes will increase the frictional forces exerted by the straps on the CFRP strips. As a result, the web stiffness of the ideal I-beam (Figure 5) will also increase. In conclusion, steel wire ropes should be more efficient than stainless steel ribbons in counteracting the out-of-plane displacements of a masonry wall subjected to hammering actions during a seismic event.

**Figure 12:**
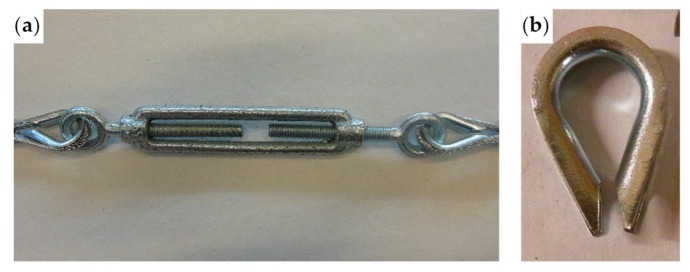
The fastening system of the second combined technique: (**a**) Two Flemish eyes connected by an eye–eye turnbuckle and (**b**) detail of the thimble placed inside a Flemish eye.

In the fastening system of the second combined technique, both loose ends of each steel wire rope form a Flemish eye (Figure 12a), which is a loop-shaped end, fixed back on the steel wire rope. A thimble installed inside the loop (Figure 12b) prevents the steel wire rope from bending too tightly when a device connected to the loop concentrates the load on a small contact area. Furthermore, the thimble protects the steel wires on the inside of the loop from abrading and pinching.

In order to fix the loose end of the loop, it is possible to use either clips or ferrules, or a combination of the two (Section 3.4). Both the clips and the ferrules are useful for avoiding fraying of the loose ends, which is a very common occurrence in steel wire ropes.

The fastening system is completed with an eye–eye turnbuckle (Figure 12a), which closes the loop by connecting the two Flemish eyes of the same steel wire rope together. The eye–eye turnbuckle is the device of the fastening system that allows us to tension the steel wire ropes. In fact, the rotation of the metal frame brings the two threaded eyebolts closer or further apart, without twisting the attached ends of the steel wire rope. This provides an adjustable pre-tension to the loop-shaped steel wire rope. Therefore, even the second combined technique is an active strengthening technique, which means that it does not require any damage to start working.

Lastly, if we choose to use stainless steel wire ropes for the straps, it is possible to avoid corrosion and compatibility problems as for the first combined technique.

Compared with the first combined technique, the second combined technique has the advantage of being adjustable. In fact, with the second combined technique it is possible to reposition the straps, without having to cut the steel wire ropes. It is also possible to adjust the tension in a steel wire rope after pre-tensioning an adjacent steel wire rope, which inevitably changes the stress in the previously tensioned steel wire rope. This makes the strapping process of the second combined technique more flexible than the strapping process of the first combined technique.

**Figure 13:**
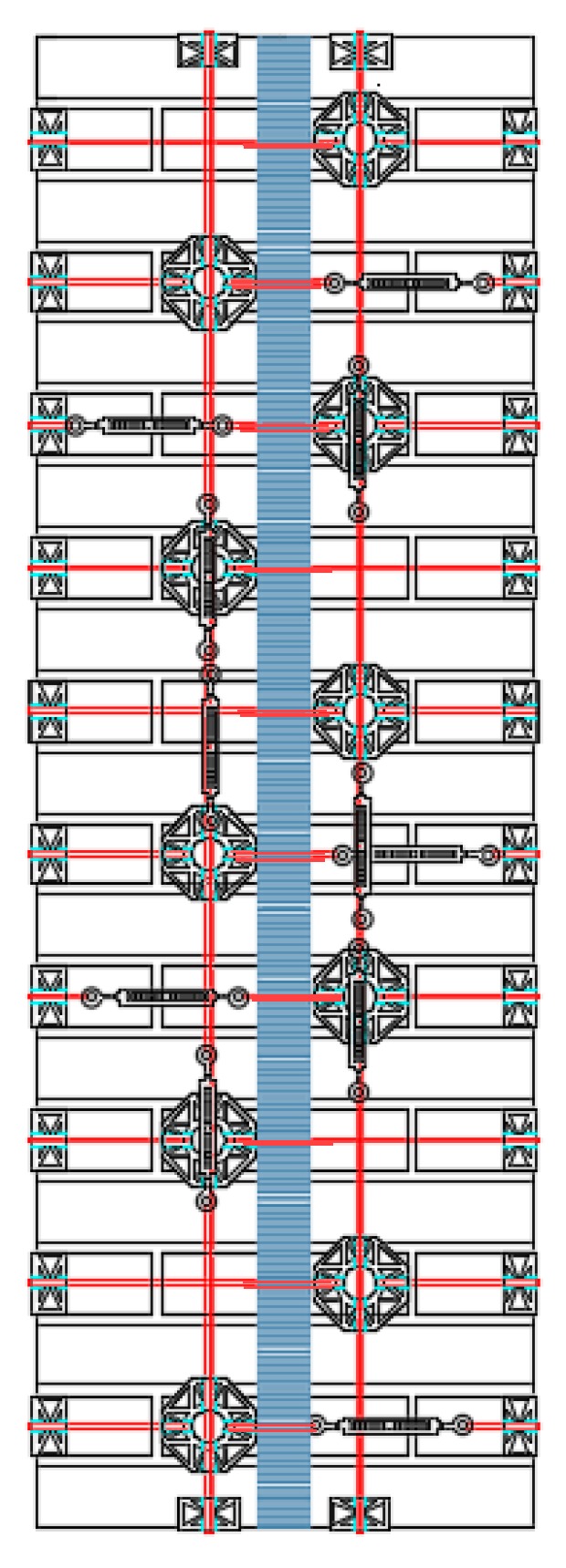
Layout scheme of the straps for the second combined technique.

Section 3 will present the results of the first experimental program carried out on the second combined technique. As for Specimens W3 and W4 of the first combined technique (Section 2.1), the experimentation consisted of performing a three-point bending flexural test on a wall specimen strengthened with straps and CFRP strips, positioned so as to obtain an I-beam behavior from the mechanical coupling between the two techniques (Figure 13). For comparison purposes, the materials and dimensions of the masonry wall and the layout of the straps are the same as those used for the first combined technique (Figure 13). In particular, the holes follow the quincunx pattern—as for Specimens W3 and W4 (Section 2.1)—and belong to intersecting three-dimensional nets.

For the strapping scheme shown in Figure 13, the horizontal straps that wrap the CFRP strips are longer than the horizontal straps that only pass over bricks. The eye–eye turnbuckles of the longer horizontal straps alternate in position on the front and back face, in order to obtain a sort of strapping symmetry on the two faces. This is useful for minimizing non-symmetrical behaviors and torsional effects induced on the specimen by the strapping system, during the test.

**Figure 14:**
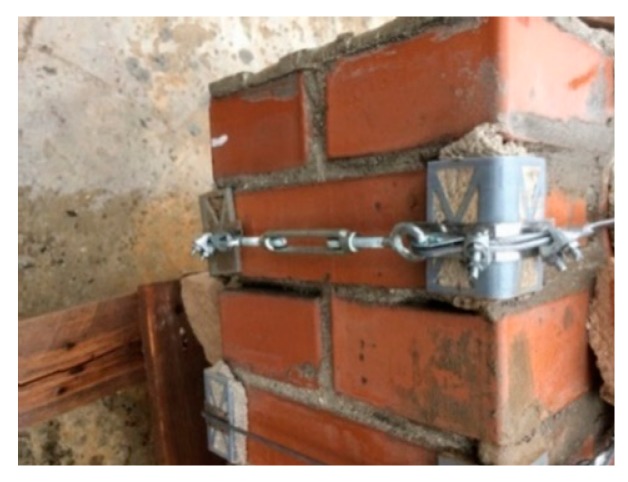
The fastening system along the lateral face of the brick wall (23 cm).

**Figure 15:**
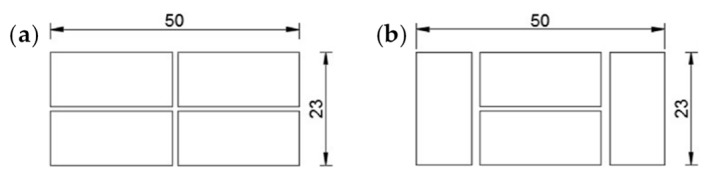
Diagram of laying the bricks for (**a**) the odd rows and (**b**) the even rows (all the measurements in cm) [1].

The eye–eye turnbuckles of the shorter horizontal straps are too long to lie on the two main faces. However, they find an optimal positioning on the lateral faces of the masonry wall, which is a two-headed wall (Figure 14), with Bolognese type bricks (24.5 × 5.5 × 11 cm). As the dimensions of the brick wall are 50 × 146 × 23 cm, the Bolognese bricks do not exactly fit the length of the wall. Therefore, it was necessary to shorten the end bricks of Figure 15a to obtain the desired length.

## 3. Experimental Program

### 3.1. Bricks and Mortar

The bricks and mortar of the experimental program are the same as those of the experimentation on the first combined technique [1]. Their mechanical characterizations complied with UNI EN 772-1 [42] and UNI EN 1015-11 [43], respectively. Table 1 and Table 2 show the results of the mechanical characterization on the bricks and the mortar, which is of the M20 type.

### 3.2. Protective Funnel-Shaped Plates and Rounded Angles

**Figure 16:**
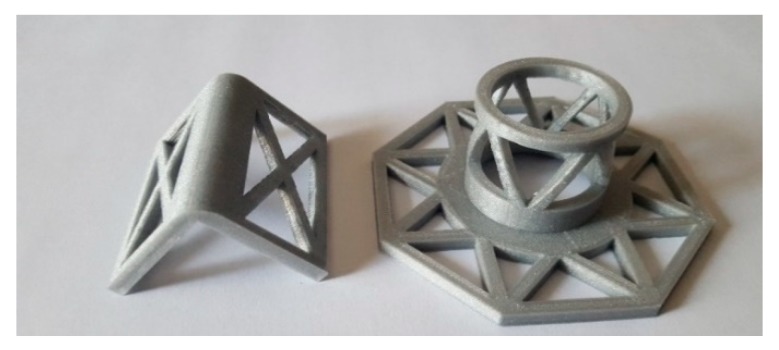
3D printed rounded angle (**on the left**) and funnel-shaped plate (**on the right**) [1].

**Figure 17:**
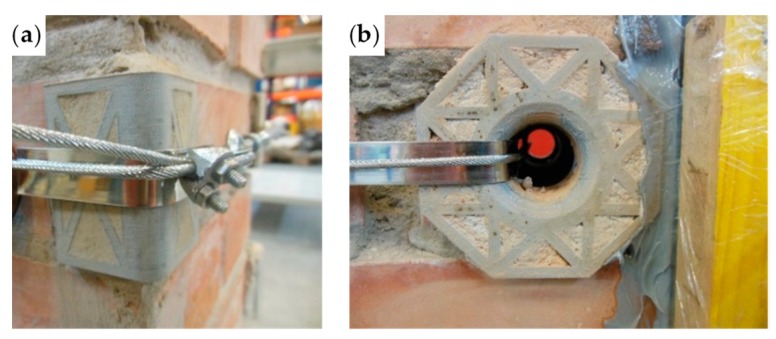
The pieces of steel ribbons positioned under the straps at the corners of the protective elements: details of (**a**) a rounded angle and (**b**) a funnel-shaped plate.

At the corners of the straps, the second combined technique uses the same protective elements as those of the first combined technique (Figure 16), that is, funnel-shaped plates and rounded angles printed with FDM (Fused Deposition Modeling), a technology that allows us to fix some intrinsic geometrical limits of hot forming. The choice of the material and the optimization of the shape of the 3D printed protective elements are part of the experimental program. For both the 3D printed plates and angles, the corners in contact with the straps are rounded, while those in contact with the edges of the wall are at 90°. Moreover, the truss shape of the flat parts increases the contact area between mortar and protective elements, improving adherence (Figure 17).

As already specified in [1], the final choice as far as the material is concerned has fallen on the PLA (Polylactic Acid) filament due to its eco-friendliness, since it is a non-toxic polyester. Although PLA is biodegradable in an exposed natural environment, its stiffness and hardness after adequate protection against degradation are similar to the stiffness and hardness of iron. The damage to the protective elements after the bending tests on the first combined technique was actually so low as to allow the reuse for the bending test on the second combined technique.

To avoid indentations, some pieces of steel ribbons protect the external corners in direct contact with the straps (Figure 17).

### 3.3. Mechanical Characterization of the Steel Wire Ropes

**Figure 18:**
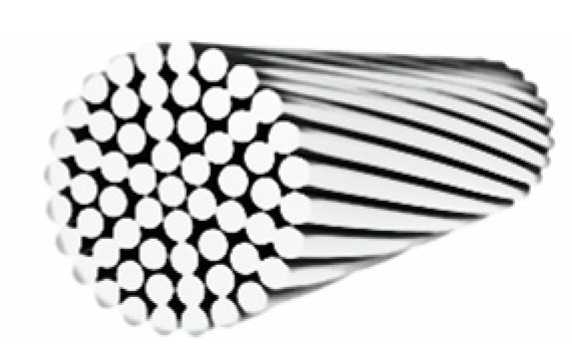
The cross-section of a spiral strand rope, as defined in EN 1993-1-11.

A steel wire rope is a set of steel wires rolled into a spiral. When a flaw occurs in a wire of a steel cable, the other wires take up the load avoiding catastrophic failures. Even internal friction is useful, in the short-term, to neutralize minor failures. However, in the long-term, friction is the main cause of rope wear. According to EN 1993-1-11 [44], the steel wire ropes of the experimentation are, more precisely, spiral strand ropes, built with independent layers of helically arranged round wires (Figure 18). The choice fell on spiral strand ropes because they are rigid and resistant to wear and corrosion [45]. These properties are suitable for static applications.

The strength of steel wires is significantly greater than the strength of structural steels. However, generally the ultimate strain of steel wires is about one-sixth of the ultimate strain of a mild steel [45].

**Figure 19:**
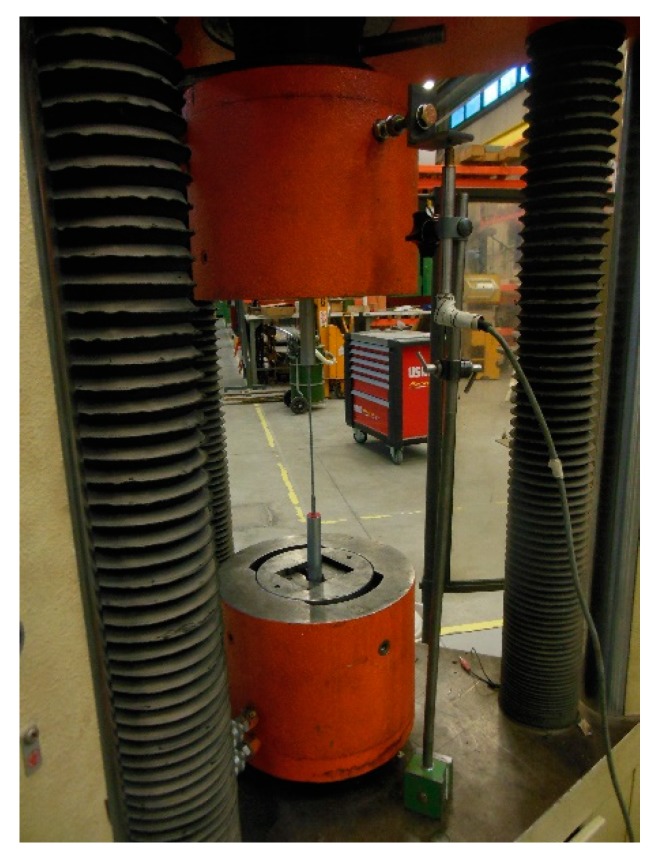
Positioning the LVDT (Linear Variable Differential Transformer) to measure the displacement of the crosshead.

ASTM A931 [46] covers the tensile testing of steel wire ropes and strands at room temperature. Figure 19 shows the experimental set-up of the tensile test performed on one spiral strand rope of the experimentation. As usual for steel ropes, an LVDT measured the crosshead displacement to obtain the extension of the specimen (Figure 19). In fact, due to high-energy specimen failures, using a traditional contacting extensometer means there is a risk of damaging and/or transforming the extensometer into a projectile. To avoid this, it is necessary to remove the extensometer prior to specimen failure. However, since the specimen will be under load, this entails a significant risk for the operator, as there is the possibility that the specimen will break during the removal of the extensometer. Furthermore, if the extensometer remains attached to the sample, reduction in rope diameter (Poisson effect) and twisting can cause the contact points to slip or the device to fall off. It could also happen that the edges of the knife cause stress concentrations, leading to premature failure.

**Figure 20:**
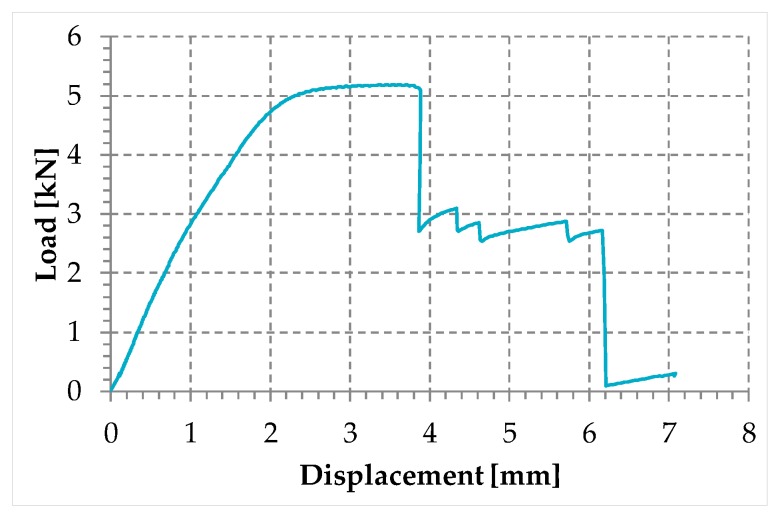
Load/displacement diagram of the steel wire rope used in the experimentation.

**Figure 21:**
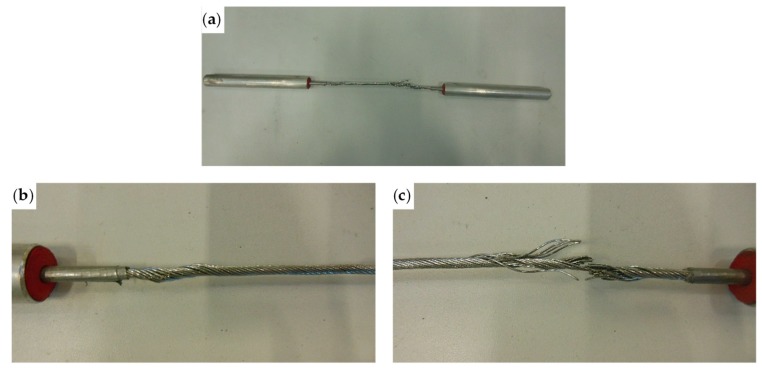
The spiral strand rope after the test: (**a**) general overview; (**b**) detail of wire twisting at the left end; and (**c**) detail of the failure due to fraying, at the right end.

Figure 20 shows the load/displacement diagram of the tested specimen, while Figure 21 shows the specimen after failure. The load drop in Figure 20 for a displacement of almost 3.9 mm corresponds to the moment in which the steel wire rope began to fray. The fraying occurred at one end of the specimen (right end in Figure 21a,c), while on the other end the individual steel wires of the specimen twisted (left end in Figure 21a,b). Each load drop in Figure 20 occurred due to the failure of one or more fraying steel wires. The first load drop did not immediately bring the sample to failure and further load increases were possible after it, as the remaining steel wires took up the load. A similar load redistribution occurred after each steel wire failure. This allowed the specimen to reach a final displacement equal to about 182% of the displacement of first fraying, with an average residual load during the fraying that is about 52% of the maximum load.

**Figure 22:**
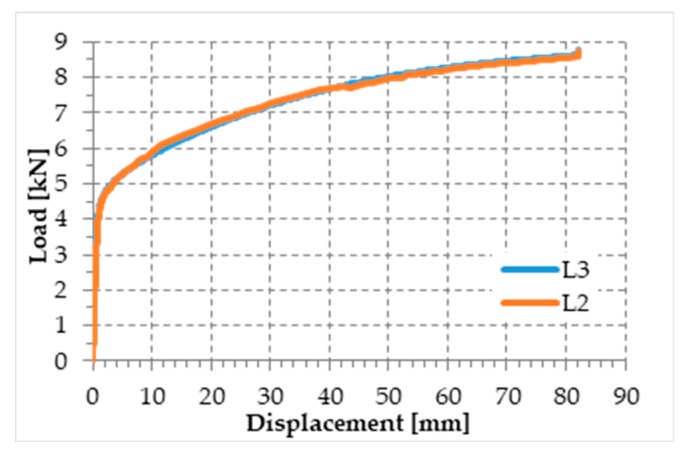
Load/displacement diagrams for the steel ribbons used in [1].

For comparison purposes, the selection criterion for the cross-section of the steel wire rope was to choose a steel wire rope with the same yield load as that of the steel ribbons used in the experimental program on the first combined technique [1]. The yield load in Figure 20 is actually comparable to the yield load of the steel ribbons of [1] (Figure 22). The specimen failure took place at the frayed end, with a final displacement equal to about 8.6% of the final displacement for the steel ribbons of [1] (Figure 22).

### 3.4. Mechanical Characterization of the Jionts

**Figure 23:**
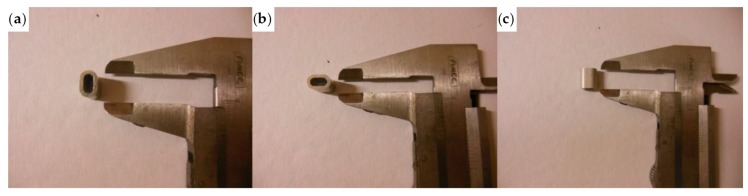
The ferrules of the experimentation: (**a**) longitudinal dimension; (**b**) transversal dimension; and (**c**) thickness.

**Figure 24:**
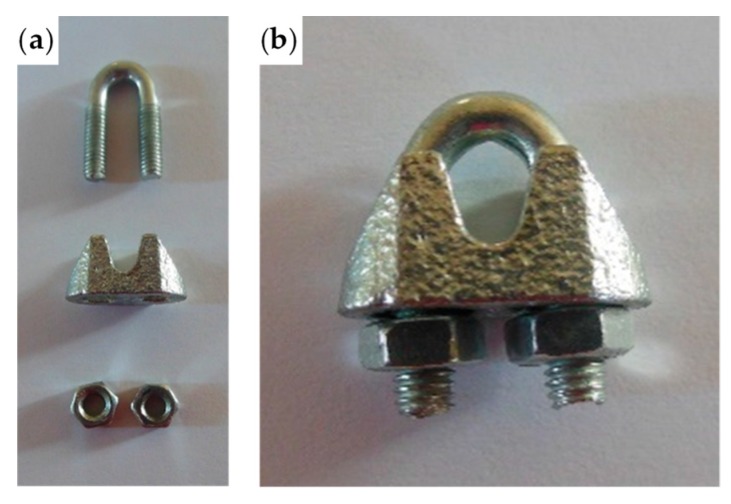
The clips of the experimentation: (**a**) constituent parts and (**b**) a clip after assembly.

As already mentioned in Section 2.2, the most common devices for fixing a Flemish eye are ferrules and clips. Ferrules—also often referred to as eyelets or grommets—are narrow circular clamps made from metal (Figure 23). They are useful to hold together and connect steel wires, generally by crimping, swaging, or otherwise deforming the ferrule to tighten it permanently on the parts it holds. A clip is a steel wire rope clamp consisting of a U-shaped bolt, a forged saddle with two holes to fit into the U-bolt, and two nuts to fix the arrangement in place (Figure 24).

**Figure 25:**
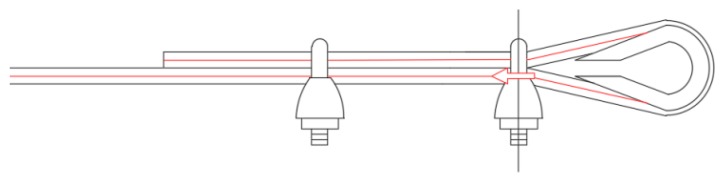
Eccentricity of the load provided by a clip to the steel wire rope, with respect to the Flemish eye.

The loose end and the steel wire rope on which to fix it pass in the space between the U-bolt and the saddle, placed one on the other on the saddle (Figure 25). By screwing the nuts, the U-bolt and the saddle approach each other, holding the two parts of the steel wire rope together.

**Figure 26:**
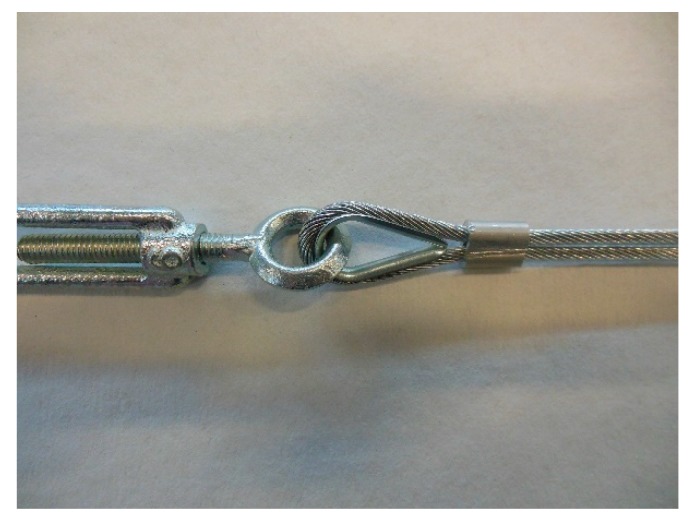
Specimen 1: Flemish eye closed by 1 ferrule.

**Figure 27:**
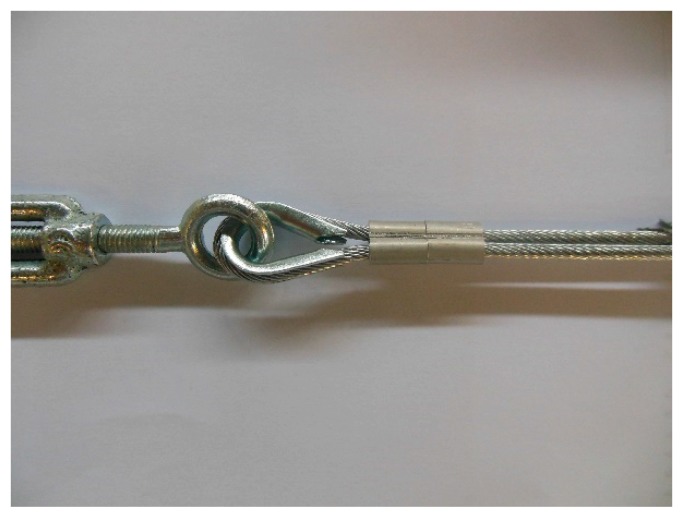
Specimen 2: Flemish eye closed by 2 ferrules.

**Figure 28:**
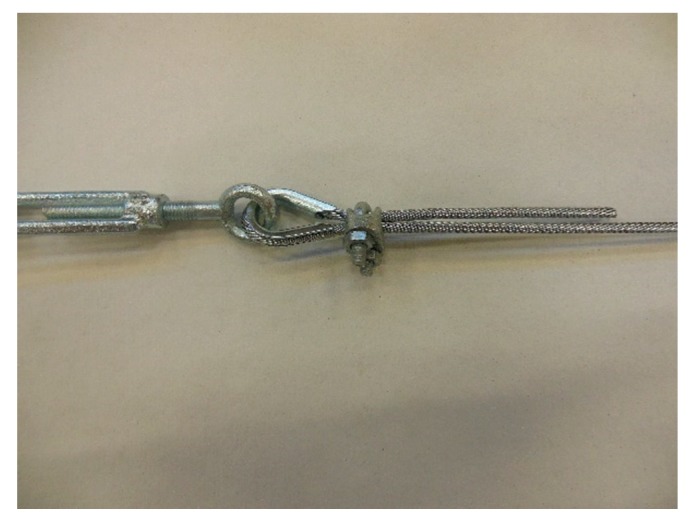
Specimen 3: Flemish eye closed by 1 clip.

**Figure 29:**
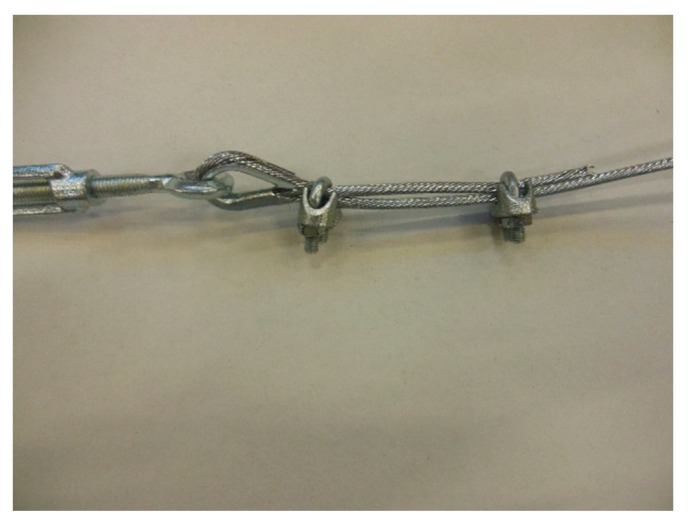
Specimen 4: Flemish eye closed by 2 clips.

**Figure 30:**
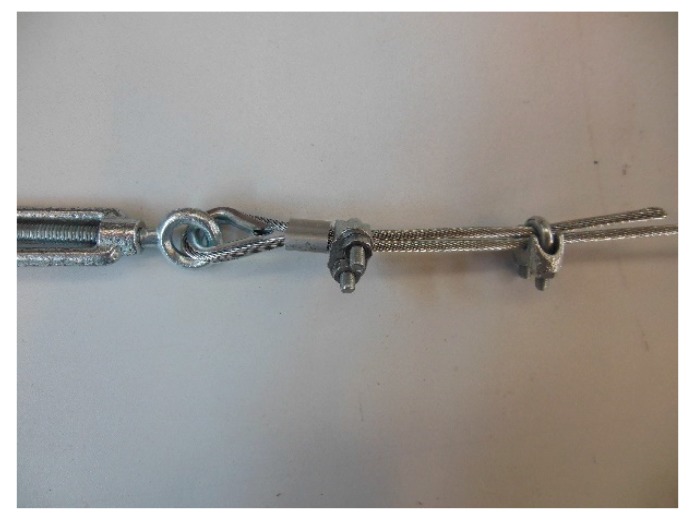
Specimen 5: Flemish eye closed by 1 ferrule and 2 clips, in succession.

**Figure 31:**
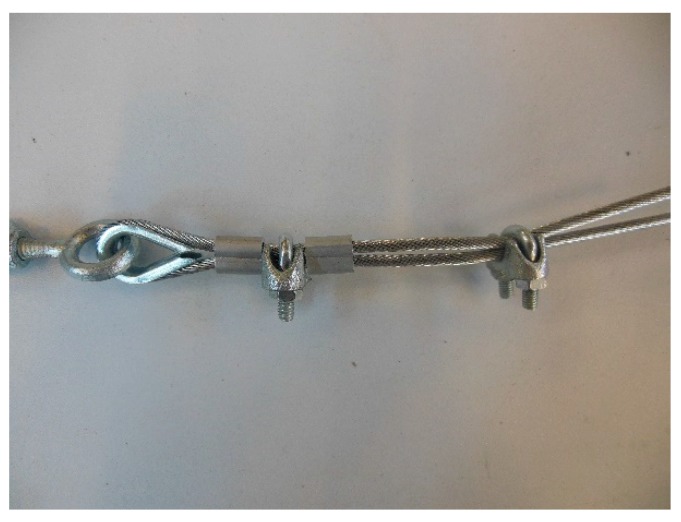
Specimen 6: Flemish eye closed by 1 ferrule, 1 clip, 1 ferrule, and 1 clip, in succession.

**Figure 32:**
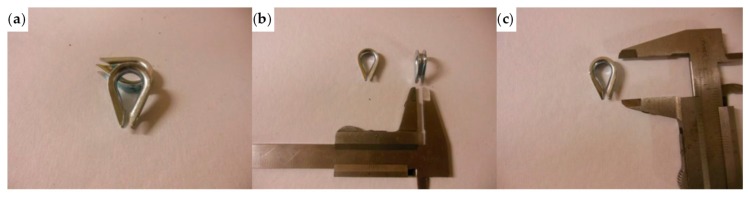
The thimbles of the experimentation: (**a**) overview; (**b**) thickness; and (**c**) longitudinal dimension.

The strength of a strap with a junction is always lower than that of the strap. Since there is no indication in the literature on the best performing joint for a steel wire rope, the experimental program included preliminary traction tests for various possible methods to close the Flemish eyes:

- 1 ferrule (Specimen 1, Figure 26);
- 2 ferrules in succession (Specimen 2, Figure 27);
- 1 clip (Specimen 3, Figure 28);
- 2 clips in succession (Specimen 4, Figure 29);
- 1 ferrule and 2 clips, in succession, starting from the Flemish eye (Specimen 5, Figure 30);
- 1 ferrule, 1 clip, a second ferrule, and a second clip, in succession, starting from the Flemish eye (Specimen 6, Figure 31);

The thimbles used to shape the Flemish eyes for the specimens listed above have the geometrical characteristics shown in Figure 32.

Specimens 1 and 2 had a very bad behavior, because they failed for a load value much lower than the maximum load of the steel wire rope without a joint (Table 3). Moreover, both specimens showed an excessive deformation of the Flemish eyes, which squashed despite the use of the thimble. However, since the maximum load of Specimen 2 was more than double the maximum load of Specimen 1 (Table 3), the use of a second ferrule helps the joint to withstand higher loads.

**Figure 33:**
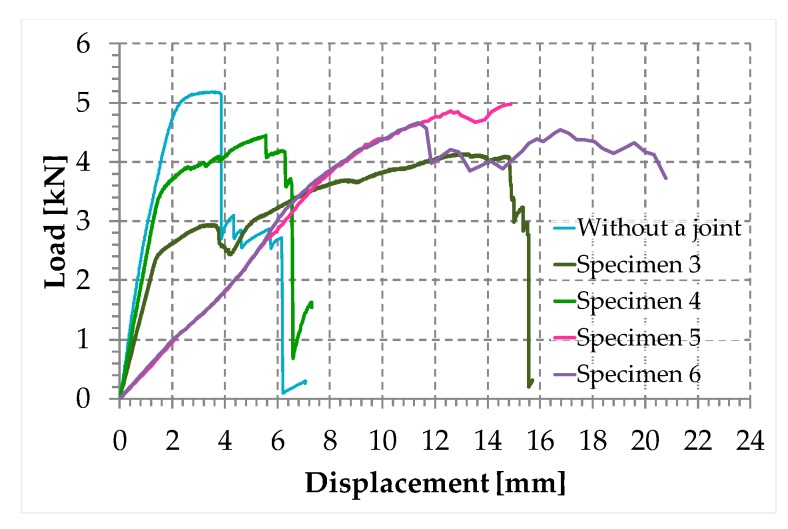
Load/displacement diagrams for 5 of the 7 specimens used for the mechanical characterization of the joints.

Figure 33 shows the comparison between the load/displacement diagrams of the steel wire rope without a joint and the four specimens with Flemish eyes closed by means of clips or combinations between clips and ferrules.

**Figure 34:**
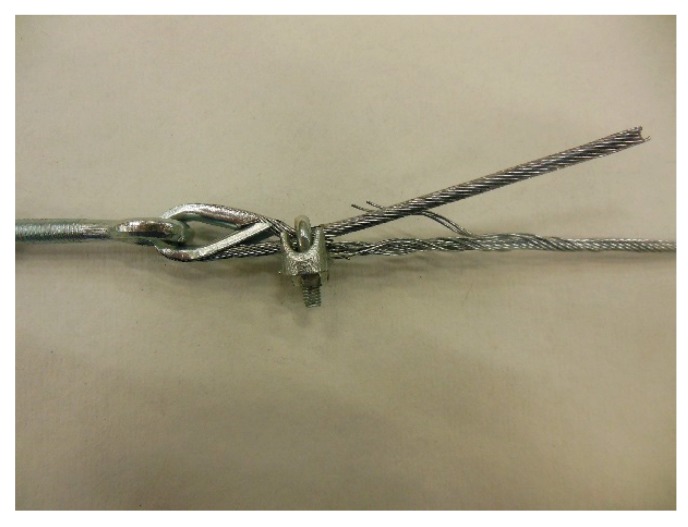
Failure mechanism of Specimen 3.

**Figure 35:**
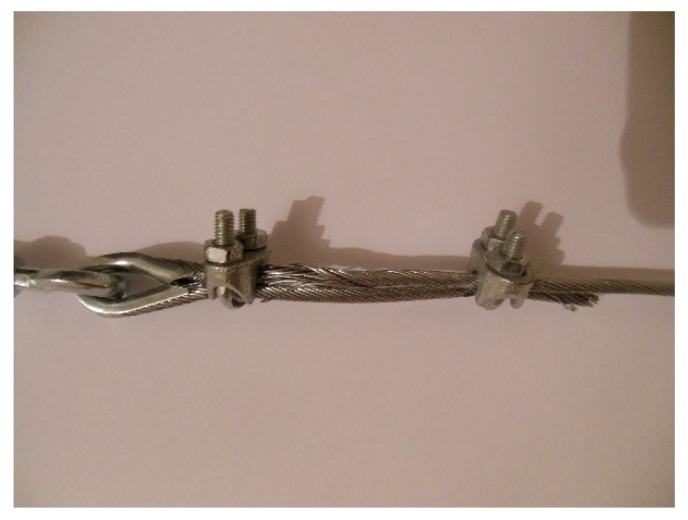
Failure mechanism of Specimen 4.

**Figure 36:**
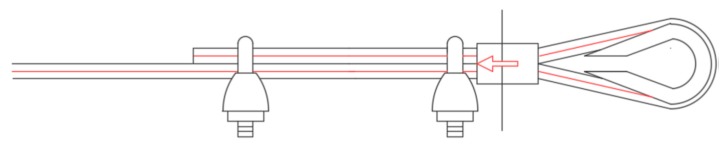
How the ferrule modifies the load centering on the two clips, compared with the load transfer scheme of Figure 25.

**Figure 37:**
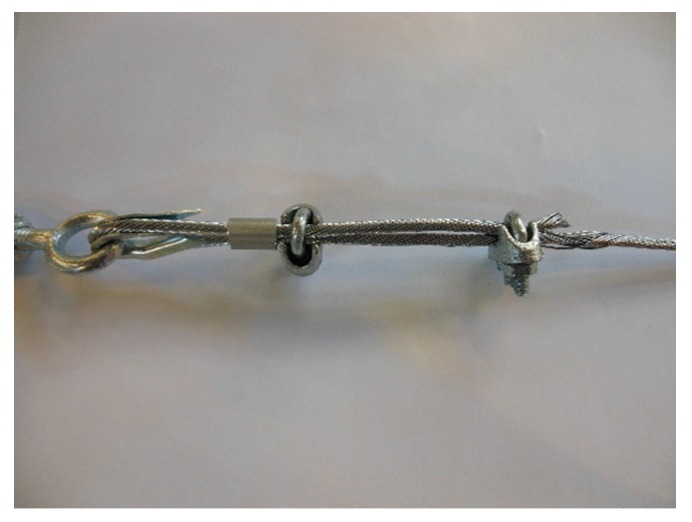
Failure mechanism of Specimen 5.

**Figure 38:**
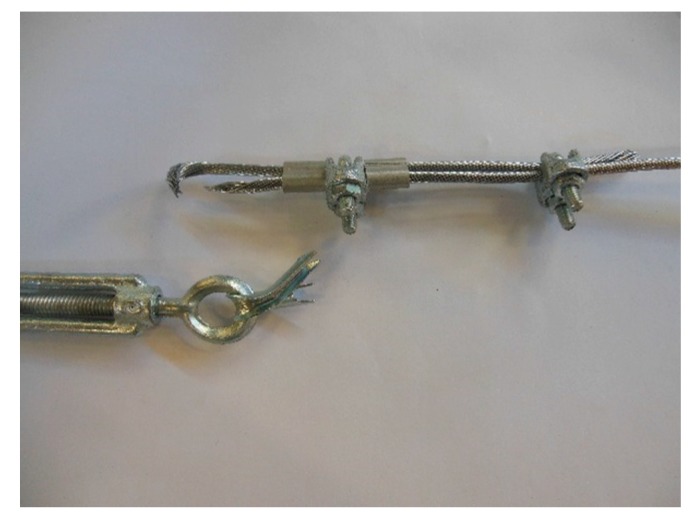
Failure mechanism of Specimen 6.

In particular:

- The load of Specimen 3 increased linearly up to a value of about 2.4 kN. Then, the slope of the load/displacement diagram decreased due to the yield behavior of the steel wires and the load continued to increase monotonically up to a value of about 2.9 kN. At this point, the specimen suffered a load drop due to the squashing of the Flemish eyes. Once the squashing of the Flemish eyes was over, the load started to rise again, up to its maximum value. Afterwards, the fraying of the steel wires quickly led the specimen to failure. It is worth noting that the fraying started from one of the two clips used to close the Flemish eyes (Figure 34). In fact, the eccentricity of the load supplied to the steel wire rope—due to the non-perfect coaxiality between the turnbuckle and the “live” side—caused the flat bearing seat of the clip to rotate, until its edge came into direct contact with the steel wires. This caused the pinching and abrading of the steel wires and, consequently, their failures in rapid succession.
- The linear behavior of the load/displacement diagram of Specimen 4 terminated for a load value of about 3.4 kN, which corresponds to approximately 142% of the load at the end of the linear branch of Specimen 1. The yield behavior of the steel wires and squashing of the thimbles took place simultaneously from this moment forward, decreasing the slope of the load/displacement diagram but without causing any load drop. The slope of the linear branch is greater than the slope of the linear branch for Specimen 3, which means that Specimen 4 is stiffer than Specimen 3. Actually, the stiffness of Specimen 4 is comparable to the stiffness of the steel wire rope without a joint. The yield behavior and squashing processes terminated with the fraying of the steel wires, which is responsible for the “step behavior” of the last part of the load/displacement diagram: Each load drop in this final part is the consequence of the failure of one or more steel wires. The fraying started from a clip, the first from the Flemish eye (Figure 35). As for Specimen 3, the cause for this lies in the non-perfect coaxiality between the turnbuckle and the “live” end (Figure 25). However, the second clip—that forces the part of the “dead” side between the two clips to bear load—partially eliminates the torsion of the first clip, delaying the fraying. This could also be the reason for the greater stiffness and maximum load of Specimen 4.
- The purpose of the fifth fastening scheme was to eliminate the torsion of the first clip of the fourth fastening scheme, that is, the clip closest to the Flemish eye. In other words, the function of the ferrule was to center the load on the two clips (Figure 36). Specimen 5 did not actually fray near the first clip (Figure 37): It frayed near the second clip. The improved load centering allowed the specimen to withstand a higher ultimate load, comparable to the ultimate load of the steel wire rope without a joint. However, the ferrule caused an excessive deformation of the Flemish eyes, as for Specimens 1 and 2. This greatly reduced the stiffness of the specimen.
- The sixth fastening scheme introduces an additional ferrule between the two clips to eliminate even the rotation of the second clip, with the aim of preventing the specimen from fraying near both clips. The second ferrule actually further improved the load centering, eliminating fraying near both clips. However, this concentrated the deformation phenomena on the thimble that twisted, cutting off the steel wires (Figure 38). The twisting of the thimble occurred due to the excessive squashing of the Flemish eye. In fact, once the two ends of the thimble come into contact, the further squashing of the Flemish eye is possible only by forcing the two ends of the thimble to slide one over the other in the direction orthogonal to the load. This causes the twisting borders of the thimble to cut the steel wires. Even in Specimen 5 the excessive deformation of the Flemish eye caused a twist of the thimble (Figure 35), but this did not lead to damage to the steel wires. Lastly, the concentration of the deformations on the Flemish eyes greatly reduced the stiffness of Specimen 6, as for Specimen 5.

In conclusion, it seems that the clips allow a certain degree of sliding between the two sides of a steel wire rope, while the ferrules are much more effective in counteracting the sliding. The sliding allowed by a clip is mainly responsible for the rotation of the clip and the consequent fraying of the steel wires that came into contact with the edge of its flat bearing seat. On the other hand, however, the elimination of the sliding by means of a ferrule causes exaggerated deformations of the Flemish eyes, which diminish the stiffness of the fastening system. The fastening system chosen for the next phase of the experimentation is the fourth fastening scheme (Figure 29) because, for the purposes of experimentation, the fastening system must be as stiff as possible.

### 3.5. Three-Point Bending Flexural Test on a Masonry Specimen

#### 3.5.1. Preparation of the Specimen and Test Setup

In order to verify the ability of the second combined technique to act as both a reinforcing and a restoring system, the wall specimen used in the experimentation is a specimen already tested in the experimental program on the first combined technique. In particular, the specimen of the first experimentation used for the second experimentation is Specimen W1 of [1], tested under a three-point bending load after strapping by means of two staggered three-dimensional nets of straps made of steel ribbons.

**Figure 39:**
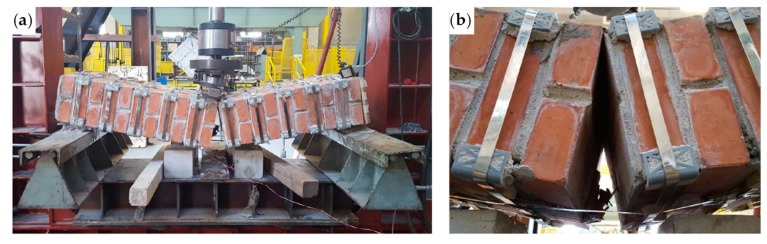
Specimen W1 shortly before the interruption of the test [1]: (**a**) disconnection mechanism and (**b**) detail of the disconnected cross-section (brick tear in the background).

During the three-point bending test on Specimen W1, an inner hinge opened near the middle cross-section (10th mortar bed joint from the right, in Figure 39a). The disconnection along the failed mortar bed joint led the two parts of the specimen to rotate around the inner hinge in a controlled manner, because the high ductility of the steel ribbons (Figure 22) allowed the disconnection to open up considerably (Figure 39), without ever causing loss of equilibrium. No straps broke during the opening of the disconnection. However, of the two longitudinal straps that crossed the disconnection, the one that passed through the hole closest to the inner hinge broke the protective funnel-shaped element and ripped off the thin layer of brick located between the disconnection and the cavity for the passage of the straps (Figure 39b).

**Figure 40:**
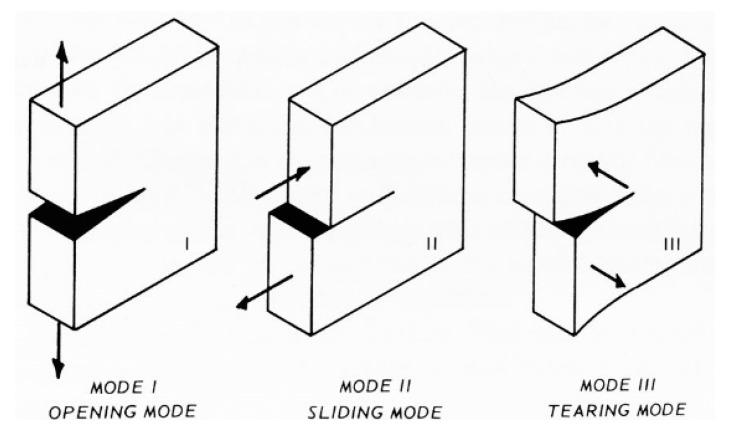
Modes of fracture.

**Figure 41:**
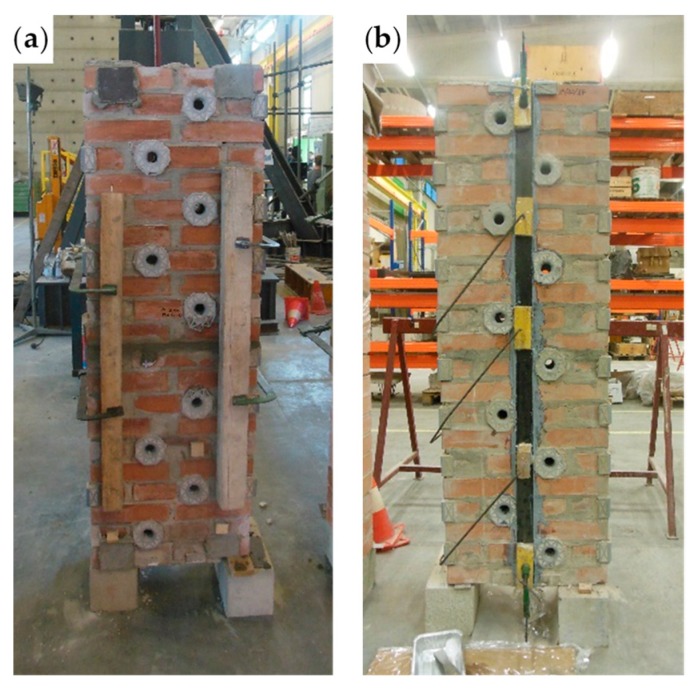
Preparation of the specimen: (**a**) restoration of the 10th mortar bed joint from below (front view) and (**b**) application of the CFRP strips after the maturing period (back view).

**Figure 42:**
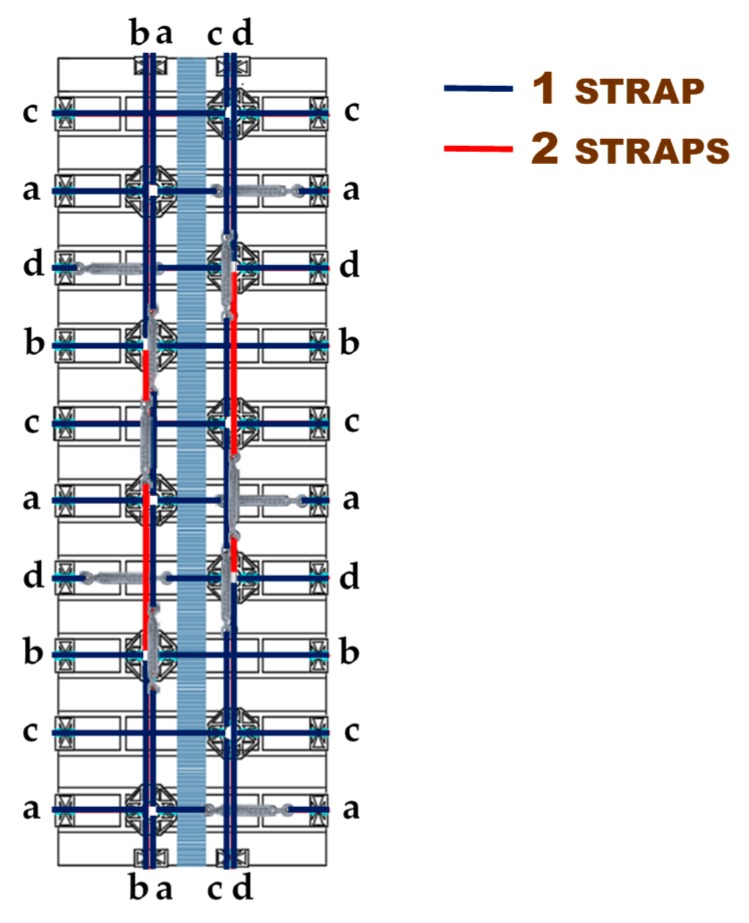
The four staggered meshes to strap the restored specimen (Specimen W5).

The preparation of the wall specimen took place as follows:

- Removal of the specimen from the testing machine;
- Removal of all the straps from the specimen;
- Cleaning of the disconnected mortar bed joint (10th mortar bed joint from the right, in Figure 39a), where the crack propagated in Mode I [47,48,49] (Figure 40);
- Overturning in the vertical configuration of the two parts resulting from the failure;
- Restoration of the cavity for the passage of the straps near the disconnected cross-section, inserting a steel tube of the same external diameter as the drilled holes;
- Restoration of the disconnected mortar bed joint (Figure 41a), after lifting and holding of the upper part of the specimen in position with a girder crane;
- Maturing of the mortar on the restored mortar bed joint;
- Application of longitudinal CFRP strips (50 mm × 1.2 mm), on both main faces of the restored specimen (Figure 41b);
- Application of the straps on the restored specimen (Figure 42).

The length of the fastening system made it impossible to pass the vertical loops in Figure 42 through adjacent holes. This made it necessary to use the holes alternately along the vertical direction. In order not to leave any unused holes, the vertical loops were staggered along the vertical direction, giving rise to the four staggered meshes (a, b, c, and d) in Figure 42. Furthermore, the number of steel wire ropes for the central vertical loops of meshes b and d were increased from 1 to 2 (Figure 42).

**Figure 43:**
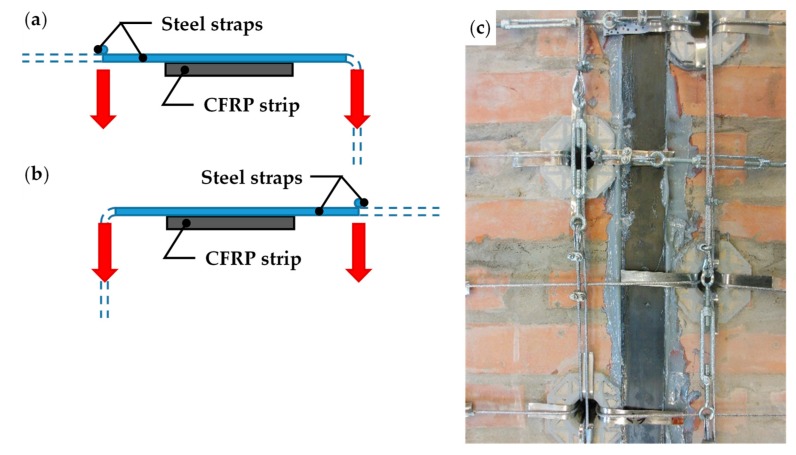
Load patterns of the CFPR strips: (**a**) cross-section view of the longitudinal strap on the left of the CFRP strip (not to scale); (**b**) cross-section view of the longitudinal strap on the right of the CFRP strip (not to scale); and (**c**) scheme sequence a, b, a, b on the strapped specimen.

As already done for the first combined technique, the strapping took place in two phases, arranging the transverse straps (parallel to the shorter sides), first, and the longitudinal straps (parallel to the longer sides), subsequently. As a result, the longitudinal straps pass over and press the transverse straps against the wall (Figure 43). This gives rise to the alternate patterns (a) and (b) of Figure 43, which allow the transverse straps to act on the CFPR strips with symmetric loads. The frictional forces caused by contact then couple the transverse straps and the CFRP strips mechanically, establishing the I-beam behavior described in Section 1.

After restoration and strengthening by the second combined technique, the new label of Specimen W1 is “Specimen W5”.

**Figure 44:**
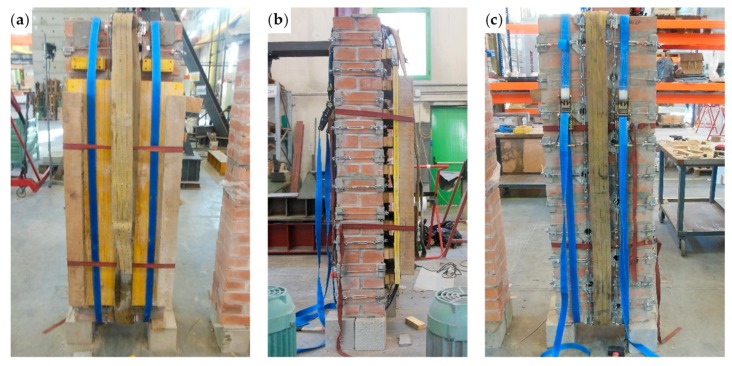
Slinging of Specimen W5, used to allow handling and overturning of the specimen by means of the girder crane: (**a**) front view; (**b**) viewed from the left; and (**c**) back view.

The bending test took place with the specimen in the horizontal configuration. This required slinging the specimen as shown in Figure 44, to allow the girder crane to hook, lift, and overturn the masonry wall.

The slings also had the function of tying a wooden “stretcher” on the front side of the masonry wall (Figure 44a), to avoid damage to the specimen during the subsequent handling phase. Some wooden spacers between the wooden “stretcher” and the masonry wall (Figure 44b) prevented the wooden “stretcher” from touching the straps. In the absence of spacers, the wooden “stretcher” could have crushed the straps when the slings pushed it against the wall, damaging the tying system.

**Figure 45:**
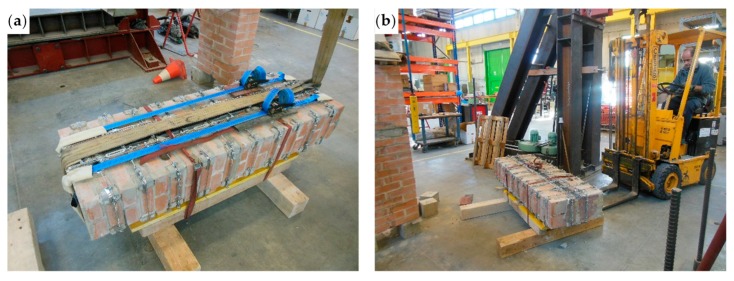
Handling of Specimen W5: (**a**) overturning of the specimen on the wooden beams, with the wooden “stretcher” placed below and (**b**) positioning the forklift forks to lift the specimen.

The overturning led to placing the wooden “stretcher” on the lower side of the specimen (Figure 45a). Furthermore, the girder crane overturned Specimen W5 on two wooden beams positioned along the shorter sides of the specimen, near the ends (Figure 45a), to leave some space under the specimen for the passage of the forklift forks (Figure 45b). When the forklift lifted Specimen W5 to place it on the testing machine, the stiffness of the wooden “stretcher” prevented the specimen from bending.

**Figure 46:**
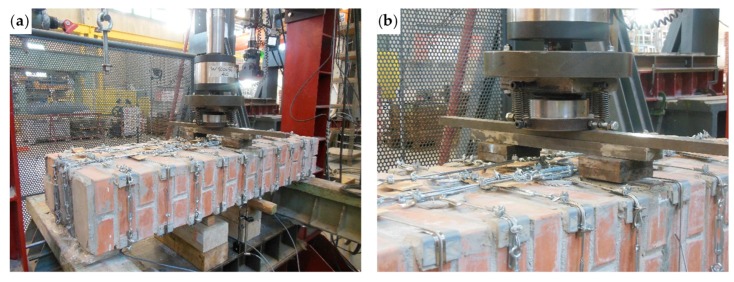
The flat steel bars for load distribution on the middle cross-section: (**a**) overview of the loading system and (**b**) detail of the passage of the steel wire ropes under the loading piston.

Some flat steel bars allowed us to distribute the load along the middle cross-section, without compressing the straps and the upper CFRP strip (Figure 46). The flat steel bar system in Figure 46 is stiff enough to provide a uniform load on the contact areas. However, the stress field induced in the specimen may not be as desired. In fact, recent experimental and analytical studies on static contact [50,51,52,53,54] do not allow us to exclude the existence of perturbative effects that do not depend on the stiffness of the loading system. As the perturbative effects concentrate along the contours of the contact areas, they can be responsible for some local damage near the contact areas.

As already done for the experimentation on the first combined technique, some LVDTs acquired the absolute displacements on the lower face of the specimen and, precisely, at the central point and the ends.

#### 3.5.2. Results and Discussion

**Figure 47:**
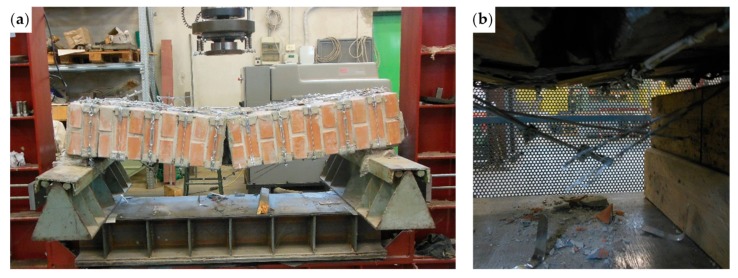
The specimen on the testing machine after the test: (**a**) front view and (**b**) detail of the broken straps under the middle cross-section (back view).

**Figure 48:**
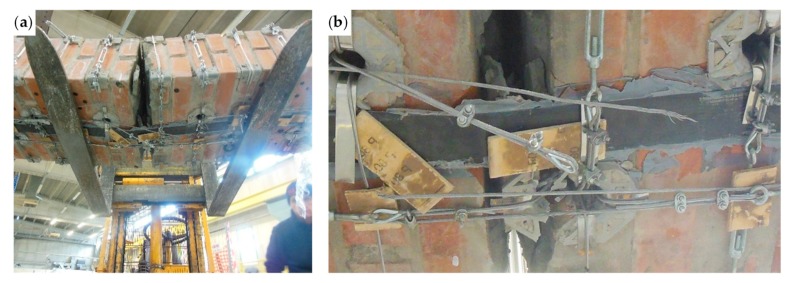
The specimen on the forklift after the test: (**a**) bottom/back view (the vertical white marks indicate the middle cross-section) and (**b**) detail of the disconnected cross-section viewed from below, with the Flemish eye that came out of the threaded eyebolt in the foreground.

The bending test took place under displacement control. The behavior of the specimen was highly ductile, as for the specimens strengthened with the first combined technique (Section 2.1 and Section 3.5.1). In fact, even in this latter case the specimen did not undergo an actual collapse because the steel wire ropes allowed the inner hinge to provide relative rotations in a controlled manner, preventing the specimen from falling (Figure 47a). However, contrary to what happened with the first combined technique (Section 3.5.1), several longitudinal straps suffered fraying and failure during the test, in particular those positioned on the middle cross-section (Figure 47b). This occurred due to the lower ductility of the steel wire ropes compared to the steel ribbons of the first combined technique (Section 3.3). In addition, one of the two threaded eyebolts of a defective turnbuckle positioned near the middle cross-section opened, due to the high load supplied by the steel wire rope. This led the Flemish eye to come out of the threaded eyebolt, interrupting the continuity of the strap (Figure 47b and Figure 48b).

The view from below in Figure 48b also shows a broken funnel-shaped plate near the middle cross-section. Since the internal disconnection of Specimen W5 opened in the same position as for Specimen W1 (Specimen W5 before the restoration), for Specimen W1 the funnel-shaped plate near the middle cross-section also broke into two parts. In Specimen W5, however, the failure of the funnel-shaped plate did not entail the interruption of the chain made by the longitudinal straps (Figure 48b), since the steel tube inserted to restore the passage of the straps (Section 3.5.1) prevented the longitudinal strap on the left of Figure 48b from tearing the brick.

**Figure 49:**
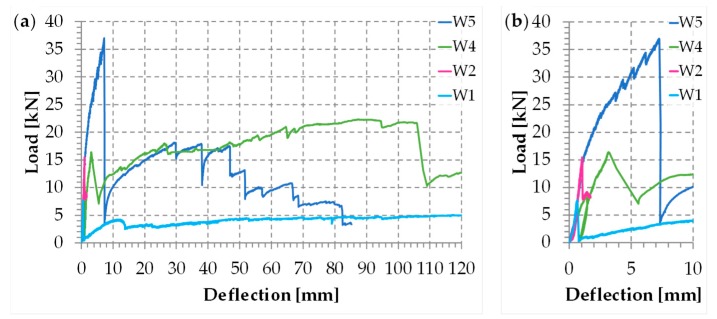
Load/deflection diagrams of the specimens reinforced with CAM-like ribbons (Specimen W1), CFRP strips (Specimen W2), first combined technique (Specimen W4), and second combined technique (Specimen W5): (**a**) complete diagrams up to the end of the tests and (**b**) detail of the peak of delamination of Specimen W5.

The maximum load of Specimen W5 is much higher than the maximum load of the same specimen before the restoration (Specimen W1): To be precise, it is 479% of the maximum load reached before the restoration (Figure 49). Therefore, the use of steel wire ropes for the straps provided a successful restoration.

**Figure 50:**
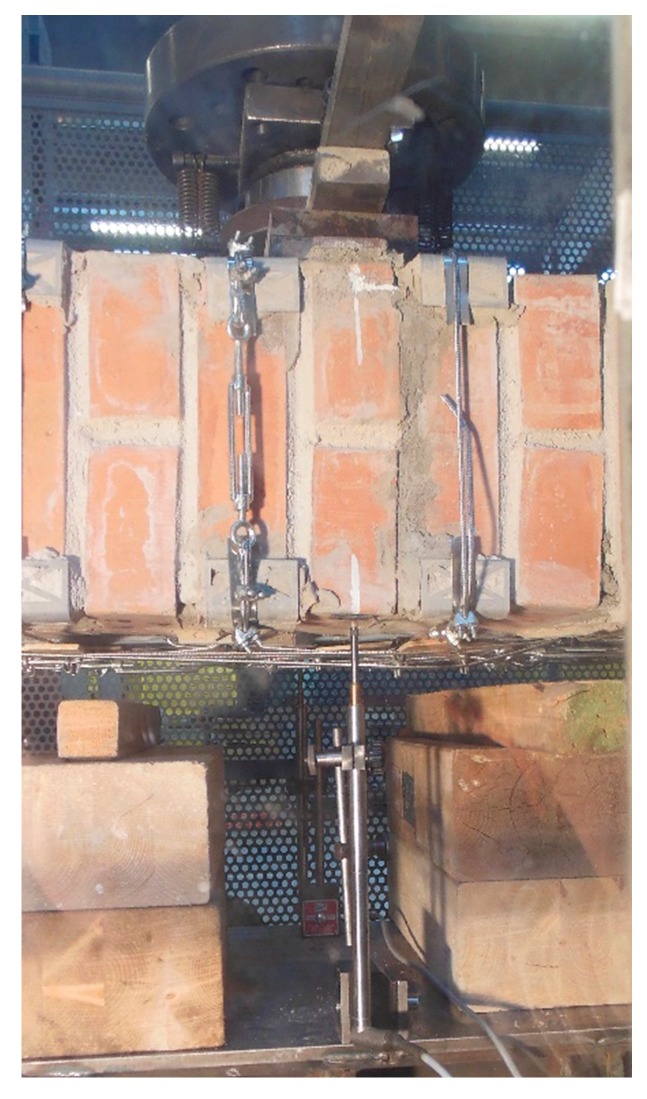
A snapshot of when the disconnection became visible for the first time (load value of about 27.122 kN), on the right of the middle cross-section: the stiffness of the longitudinal straps does not allow free rotation around the inner hinge (back view, the vertical white marks indicate the middle cross-section).

The internal disconnection occurred for a load value of about 23 kN. This disconnection did not result in any appreciable load drop in the load/deflection diagram of Specimen W5, but led to a slight decrease in the slope of the diagram (Figure 49). Moreover, the disconnection was not appreciable to the naked eye at first, since the straps took up the load no longer supported by the failed mortar, not allowing the disconnection to open. This increased the load on the longitudinal straps positioned above the disconnected cross-section. As a result, some steel wire ropes began to fray. The four load drops for the load values of about 27.122, 29.520, 31.698, and 34.444 kN (Figure 49b) occurred precisely because of the fraying. After each of these load drops, the load started to rise again, exceeding the load reached before fraying. Furthermore, the first load drop made the disconnection visible along the mortar bed joint (Figure 50).

At the load value of 36.992 kN (maximum load), a fifth load drop occurred (Figure 49). This additional load drop has a different cause than the previous ones, as it is the consequence of the delamination of both CFRP strips. It is worth noting that the load of delamination of Specimen W5 is about 239% of the delamination load with only the CFRP strips (Specimen W2 of [1], see Figure 49). This is a significant improvement compared to the first combined technique (Specimen W4 of [1], see Figure 49), which provided a delamination load equal to 106% of the delamination load of Specimen W2. The improvement also concerned the ductility up to delamination of the specimen reinforced with the second combined technique: The delamination deflection of Specimen W5 is approximately 242% of the delamination deflection of Specimen W4 and 743% of the delamination deflection of Specimen W2.

The second combined technique is more performant than the first combined technique also as far as the stiffness of pre-delamination is concerned. In particular, the stiffness of Specimen W5 is almost equal to the stiffness of Specimen W2 up to the delamination load of Specimen W2 (Figure 49b). Specimen W4, on the contrary, suffered a decrease in stiffness starting from a load value equal to about 30% of the delamination load of Specimen W2 (Figure 49b).

**Figure 51:**
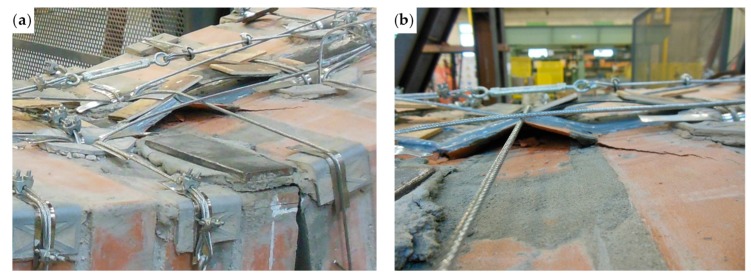
Buckling of the upper CFRP strip: (**a**) how the transverse and longitudinal steel wire ropes hold back the upper CFRP strip, counteracting delamination on the middle cross-section and (**b**) detail of the brick peeling caused by delamination.

Since the delamination of the upper CFRP strip occurs due to buckling, the detachment of the upper CFRP strip was in Mode I (Figure 40), near the disconnected cross-section. As usual for detachments in Mode I between CFRP strips and masonry [55], delamination caused the tearing of a thin layer composed by brick and mortar (Figure 51b), since the substrate is the element with the lower tensile strength in the system consisting of CFRP, resin, and masonry.

Even on the lower side, the delamination caused the tearing of a thin layer of substrate (Figure 48b), although the failure mode on the lower face of a bended specimen occurs in Mode II (Figure 40). Actually, the substrate is the weakest element also for shear sliding on mortar bed joints reinforced with CFRP strips [56,57,58], since the substrate is the component with the lower shear strength in the CFRP/resin/masonry system.

The significant decrease in load after the maximum load of Specimen W5 is a measure of the release of high energy that characterized delamination. However, as shown in Figure 49, the load of post-delamination began to rise to such an extent as to exceed the delamination load of Specimen W2. Actually, after the load recovery of post-delamination, the load/deflection diagram of Specimen W5 is almost superimposable to the load/deflection diagram of Specimen W4, at least up to the deflection value of about 47 mm. The superimposition between the two diagrams in the post-delamination phase indicates that the chosen type and amount of steel wire ropes are capable of bearing the same load of the steel ribbons of Specimen W4.

The load recovery of post-peak is a consequence of the combined action of the transverse and longitudinal straps on the CFRP strips. As far as the upper CFRP strip is concerned, Figure 51 shows how the combined action of the straps led the upper CFRP strip to delaminate only in part, allowing the non-delaminated portions of the strip to continue to be part of the ideal I-beam generated by the mechanical coupling. In particular, the longitudinal steel wire rope in the foreground of Figure 51a helps the underlying transverse steel wire rope to hold back the upper CFRP strip by providing a downward force at the intersection between the straps, according to the simplified schemes of Figure 43a,b. The downward action of the longitudinal steel wire rope causes a change in the curvature of the transverse steel wire rope below (Figure 51a). However, due to the geometric effect of relative rotation caused by the inner hinge under the longitudinal steel wire rope, the downward action of the longitudinal steel wire rope would have been more significant if the holes for the passage of the longitudinal steel wire rope had been closer.

Despite the load recovering after delamination allowing Specimen W5 to overcome the delamination load of Specimen W2, it is worth remembering that a load drop must not exceed 15–20% of the delamination load to avoid overloading phenomena in adjacent structural elements. Therefore, the maximum load of Specimen W5 would probably represent the service limit of the second combined technique in a building. Consequently, the main result obtained with the second combined technique does not lie in the load of post-delamination but in having increased the delamination load by approximately 139% with respect to the delamination load of Specimen W2. However, the existence of a long post-delamination branch together with the ability of the combined technique to establish a good box-type behavior even after damage has occurred (Section 1) allow us to affirm that the second combined technique also finds a second use beyond the structural limit, as a device for safeguarding life. Actually, both combined techniques can prevent the building from collapsing even if severely damaged, protecting people from possible injury.

For the load value of about 47 mm, the fraying became no longer sustainable by the remaining steel wire ropes and the longitudinal straps started to break in slow succession. The test ended without reaching the collapse of the specimen, when the operator recognized a possible damage to the instrumentation. At the end of the test, only one of the longitudinal straps that crossed the disconnected cross-section was still resisting the load (the longitudinal strap passing through the restored hole in Figure 48b).

## 4. Conclusions

The combined technique discussed in this paper (the second combined technique) originates from the experimentation on a previous combined technique (the first combined technique), useful for increasing the out-of-plane strength of masonry walls. The first combined technique exploits friction to mechanically couple CFRP strips and straps made of stainless steel ribbons. The effect of the mechanical coupling is an ideal I-beam behavior, capable of counteracting the out-of-plane displacements of masonry walls. The experimental result at the base of the second combined technique is that a greater stiffness of the straps seems to improve the performance of the ideal I-beam. Therefore, it seemed reasonable to use stiffer straps, made of steel wire ropes, keeping the coupling technique between the straps and the CFRP strips unaltered.

After a preliminary phase that allowed the identification of the best performing fastening system for the steel wire ropes, a new three-point bending flexural test showed that the strap stiffness actually affects the behavior of the ideal I-beam. In particular, the second combined technique proved to be able to provide the reinforced specimen with greater stiffness for small deflections, when compared with the first combined technique. Actually, the stiffness of the specimen reinforced with the second combined technique is the same as that given by the CFRP strips, while the first combined technique is more deformable.

Even more interesting are the results in terms of ductility and maximum load: The wire ropes delayed the delamination, increasing both the deflection and the load at the time of delamination. Even the steel ribbons increased both the deflection and the load at the time of delamination, but to a lesser extent. In particular, the second combined technique is much more effective than the first combined technique in increasing the delamination load. This, together with the increased delamination ductility, is a remarkable achievement for the second combined technique, since any traditional strengthening system acts on a structural element increasing either strength or ductility.

The increase in stiffness for small deflections, the greater maximum load, and the greater delamination deflection indicate that the ideal I-beam mechanism improves the behavior of the structural element even before delamination. On the contrary, with the first combined technique the ideal I-beam mechanism becomes evident only after delamination. Thus, the greater stiffness of the steel wire ropes makes it possible to make better use of the advantages of the mechanical coupling permitted by friction.

It is worth noting that the mechanical coupling modifies the resistant mechanisms of the coupled strengthening systems giving rise to a new resistant mechanism. In fact, the two combined techniques offer a strengthening effect similar to that of a buttress or other similar cross-bracing devices [59]. In contrast, both constituent strengthening systems are effective in the plane of the wall, if taken individually. Consequently, the strength parameters of the combined techniques are not the average values of the strength parameters of the two constituent strengthening systems.

As a last observation, the second combined technique also offers a post-delamination contribution similar to that of the first combined technique, with partial recovery of the load after delamination. In particular, the recovered load of post-delamination exceeds the delamination load of the first combined technique. However, due to the high load drop caused by delamination, the importance of the load/deflection diagram after delamination does not lie in the recovered load but in the very existence of a post-delamination phase, which is a peculiarity of the combined techniques. In fact, this phase indicates that the I-beam mechanism survives delamination and—more importantly—the second combined technique is useful to avoid structural collapses up to high deflections. Therefore, as for the first combined technique, the second combined technique acts as both a reinforcement system and a protection device. This makes the second combined technique very effective in offering protection against damage caused by the hammering actions provided by earthquakes [60], as well as by impact [61,62,63,64,65] and blast [66,67,68,69,70].

## 5. Future Developments

The analysis of the results on the second combined technique outlined some possible improvements to make the technique even better performing:

- In order to avoid that the breakage of one or more funnel-shaped elements interrupts the chain of the longitudinal straps, it may be useful to re-design the 3D-printed elements or use a more resistant material for the protective elements.
- In order to avoid that the geometric effects due to the relative rotations around the inner hinge frustrate the action of the longitudinal straps on the transverse straps, it may be useful to decrease the length of the loops near the middle cross-section, where the inner hinge has the maximum probability of localization.
- In order to avoid that excessive post-delamination fraying leads to the collapse of the structural element, it may be useful to use longitudinal stainless steel ribbons in addition to the longitudinal steel wire ropes, at least near the middle cross-section. Since the function of the additional steel ribbons is only to safeguard life, they do not need a pre-tension.
- In order to avoid excessive load drops and dangerous release of energy at the time of delamination—which are peculiarities of epoxy resins—it may be useful to replace the epoxy resin (organic) with a mortar matrix (inorganic).

As for the last suggested improvement among those listed above, a possible solution consists in replacing the CFRP strips with TRMs (textile-reinforced mortars) [71], also often referred to as TRCs (textile reinforced concretes) [72] or FRCMs (fabric reinforced cementitious matrices) [73]. In fact, TRMs use cement or hydraulic-lime-based mortars, both of which are inorganic matrices, to impregnate high-strength fibers in the form of open-meshes, which are textiles.

The monotonic tensile tests on TRM specimens showed that the stress/strain curve of TRMs comprises three distinct linear branches [74]:

- First ascending branch: The specimen remains un-cracked.
- Horizontal branch (constant stress at increasing strains): Multiple cracks develop in the mortar, after the first cracking. During this phase, the area of the resistant cross-section of the mortar decreases progressively [75,76,77,78,79,80] due to the gradual development of the crack pattern that causes a progressive transfer of stress from the mortar to the fibers of the open-mesh textile.
- Second ascending branch, characterized by a reduced slope with respect to that of the first branch: The crack pattern has reached its maximum development and the residual stiffness is due to the fibers of the textile, up to the break.

The tests performed on the interface bond outlined different failure modes for TRMs [74]: (1) Sliding of the fibers in the matrix (the most common failure mode); (2) debonding with substrate tearing; (3) debonding in the substrate–matrix interface; and (4) TRM rupture. Depending on which of these failure modes activates at the soffit of a beam, the failure of the TRM reinforcement system under bending loading can be progressive or sudden [81]. It is precisely the first case that deserves further study in combination with the second combined technique, to avoid sudden load drops that can trigger dangerous energy releases at the time of delamination.

As a final remark, a TRM strengthening technique can also allow us to avoid some of the typical drawbacks of FRPs when using the epoxy resin as interfacial adhesive as well as bonding matrix, namely, high costs, incompatibility with substrate materials, sensitivity to high temperatures and fire, and impossibility of application on wet surfaces. Actually, TRM is low-cost, compatible with concrete and masonry, fire resistant, and usable at low temperatures or on wet surfaces.

## Figures and Tables

**Table 1:** Characteristics of the six brick specimens tested in uniaxial compression (UNI EN 772-1) [1].

Specimen	Dimensions [mm]	Weight [g]	Breaking Load [N]	Compressive Strength [N/mm^2^]	Normalized Compressive Strength [N/mm^2^]
PA1	55 × 54 × 55	296.10	116,436	39.632	34.480
PA2	57 × 57 × 55	317.80	165,730	50.911	44.293
PB1	55 × 53 × 55	297.50	146,733	49.624	43.173
PB2	56 × 55 × 57	319.20	142,681	46.099	40.106
PC1	56 × 53 × 56	310.50	144,933	47.777	41.566
PC2	56 × 55 × 56	317.10	149,422	48.148	41.888

**Table 2:** Characteristics of the mortar specimens tested according to UNI EN 1015-11/2007 [1].

Specimens of the Flexural Tests	Dimensions [mm]	Weight [g]	Breaking Load in Bending [N]	Flexural Strength [N/mm^2^]	Specimens of the Compression Tests	Breaking Load in Compression [N]	Compressive Strength [N/mm^2^]
P1	40 × 40 × 160	466.42	1758	4.120	P1A	30,530	19.080
					P1B	36,730	22.960
P2	40 × 40 × 160	469.81	1838	4.310	P2A	30,980	19.360
					P2B	30,930	19.330
P3	40 × 40 × 160	470.42	1443	3.380	P3A	27,500	17.190
					P3B	28,530	17.830
P4	40 × 40 × 160	459.63	1885	4.420	P4A	34,544	21.590
					P4B	27,730	17.330
P5	40 × 40 × 160	463.81	1990	4.660	P5A	33,880	21.180
					P5B	35,200	22.000
P6	40 × 40 × 160	462.01	1598	3.750	P6A	30,400	19.000
					P6B	30,450	19.030

**Table 3:** Maximum loads of the steel wire rope and the six jointed specimens.

Specimen	Maximum Load [kN]
Without a joint	5.186
Specimen 1	1.100
Specimen 2	2.570
Specimen 3	4.139
Specimen 4	4.447
Specimen 5	4.974
Specimen 6	4.655
